# Long-Lasting Desynchronization Effects of Coordinated Reset Stimulation Improved by Random Jitters

**DOI:** 10.3389/fphys.2021.719680

**Published:** 2021-09-24

**Authors:** Ali Khaledi-Nasab, Justus A. Kromer, Peter A. Tass

**Affiliations:** Department of Neurosurgery, Stanford University, Stanford, CA, United States

**Keywords:** coordinated reset stimulation, spike-timing-dependent plasticity (STDP), random jitter, long-lasting desynchronization, stimulation-induced decoupling

## Abstract

Abnormally strong synchronized activity is related to several neurological disorders, including essential tremor, epilepsy, and Parkinson's disease. Chronic high-frequency deep brain stimulation (HF DBS) is an established treatment for advanced Parkinson's disease. To reduce the delivered integral electrical current, novel theory-based stimulation techniques such as coordinated reset (CR) stimulation directly counteract the abnormal synchronous firing by delivering phase-shifted stimuli through multiple stimulation sites. In computational studies in neuronal networks with spike-timing-dependent plasticity (STDP), it was shown that CR stimulation down-regulates synaptic weights and drives the network into an attractor of a stable desynchronized state. This led to desynchronization effects that outlasted the stimulation. Corresponding long-lasting therapeutic effects were observed in preclinical and clinical studies. Computational studies suggest that long-lasting effects of CR stimulation depend on the adjustment of the stimulation frequency to the dominant synchronous rhythm. This may limit clinical applicability as different pathological rhythms may coexist. To increase the robustness of the long-lasting effects, we study randomized versions of CR stimulation in networks of leaky integrate-and-fire neurons with STDP. Randomization is obtained by adding random jitters to the stimulation times and by shuffling the sequence of stimulation site activations. We study the corresponding long-lasting effects using analytical calculations and computer simulations. We show that random jitters increase the robustness of long-lasting effects with respect to changes of the number of stimulation sites and the stimulation frequency. In contrast, shuffling does not increase parameter robustness of long-lasting effects. Studying the relation between acute, acute after-, and long-lasting effects of stimulation, we find that both acute after- and long-lasting effects are strongly determined by the stimulation-induced synaptic reshaping, whereas acute effects solely depend on the statistics of administered stimuli. We find that the stimulation duration is another important parameter, as effective stimulation only entails long-lasting effects after a sufficient stimulation duration. Our results show that long-lasting therapeutic effects of CR stimulation with random jitters are more robust than those of regular CR stimulation. This might reduce the parameter adjustment time in future clinical trials and make CR with random jitters more suitable for treating brain disorders with abnormal synchronization in multiple frequency bands.

## 1. Introduction

The human organism can be viewed as an integrated network where complex physiological systems continuously interact; whereby the regulatory mechanisms of one system may affect others or the organism as a whole (Bashan et al., 2012; Ivanov et al., 2016). Associations between network topology and network functionality may provide insights into how distinct pathological/physiological states emerge from nonlinear interactions between multi-compartment complex systems (Bartsch et al., 2015). On the other hand, therapeutic effects of stimulation applied to one system may spread throughout the entire network (Pfeifer et al., 2021).

In neurological disorders, such as epilepsy (Mormann et al., 2000) or Parkinson's disease (PD) (Alberts et al., 1969; Nini et al., 1995), patients suffer from pronounced motor symptoms that are caused by impaired brain activity. In PD, several strongly interconnected brain areas are involved, including the basal ganglia, the thalamus, and the sensorimotor cortex. In several of these areas, symptom-related abnormal neuronal synchrony has been observed (Nini et al., 1995; Hammond et al., 2007). An established treatment for advanced Parkinson's disease is high-frequency deep brain stimulation (HF DBS). HF DBS has been delivered to several target areas, such as the subthalamic nucleus (STN) (Krack et al., 2003) or the ventral intermediate nucleus of the thalamus (Benabid et al., 1991). HF DBS of the STN is the standard of care for treating medically refractory Parkinson's disease, however, there is no consensus on its mechanism of action (Ashkan et al., 2017; Jakobs et al., 2019; Lozano et al., 2019; Krauss et al., 2020).

Recently, DBS has been suggested as a treatment for other disorders, including obsessive-compulsive disorder (OCD) (Vicheva et al., 2020). Symptoms of OCD include uncontrollable recurring thoughts (obsessions) and repetitive behaviors (compulsions). OCD is also associated with substantial comorbidities, including substance use disorders, anxiety, and impulse-control (Ruscio et al., 2010; Vicheva et al., 2020). The exact mechanism underlying OCD and the therapeutic effect of DBS for OCD remain enigmatic (Bourne et al., 2012; Ahmari and Dougherty, 2015; Vicheva et al., 2020).

Besides several benefits, continuous HF DBS has limitations. In PD patients, HF DBS may successfully suppress symptoms while stimulation is on, however, symptoms return shortly after cessation of stimulation (Temperli et al., 2003). Permanent stimulation of the target area and surrounding tracts and nuclei as well as the corresponding medication dose adjustment may lead to DBS-induced movement disorders (Baizabal-Carvallo and Jankovic, 2016), e.g., characterized by dyskinesias, gait disorder, dysarthria, ataxia etc. (Rodriguez-Oroz et al., 2005; Temel et al., 2006; Moreau et al., 2008; Tripoliti et al., 2008; Schrader et al., 2011; Baizabal-Carvallo and Jankovic, 2016; Xie et al., 2017).

To substantially reduce the integral amount of delivered stimulation current and the risk of unwanted side effects, several studies focused on developing stimulation approaches that specifically counteract pathological synchrony. Some approaches use delayed feedback to desynchronize networks of oscillators (Rosenblum and Pikovsky, 2004a,b; Popovych et al., 2005; Hauptmann et al., 2005a,b,c; Popovych et al., 2006a,b; Pyragas et al., 2007; Popovych and Tass, 2010); clinically this could be implemented using linear or nonlinear delayed feedback envelope pulse trains (Popovych et al., 2017a,b). Other studies analyzed the nonlinear response of an ensemble of coupled oscillators to external stimuli. For instance, an ensemble of synchronized oscillators can be desynchronized by delivering a single stimulus pulse at a vulnerable phase of the collective rhythm (Mines, 1914; Winfree, 1977, 1980; Warman and Durand, 1989; Tass, 1999). During double-pulse stimulation, such a desynchronizing pulse is delivered shortly after a strong phase-resetting pulse to increase robustness (Tass, 2001, 2002; Zhai et al., 2005). Other studies suggest the delivery of periodic stimulation. It was shown that periodic stimulation with certain frequencies can desynchronize a synchronous ensemble of oscillators. This effect is known as chaotic desynchronization (Wilson et al., 2011). Phasic burst stimulation is another approach that aims at increasing the phase differences between individual oscillators by delivering stimulation bursts at certain phases of the collective rhythm. Corresponding phases were predicted using estimated phase response curves calculated for each patient. A corresponding closed-loop method was suggested in Holt et al. (2016). Other techniques deliver spatio-temporal stimulus patterns through multiple stimulation sites, one of which is coordinated reset stimulation (CR) (Tass, 2003b). During CR stimulation, desynchronization is achieved by delivering phase-shifted stimuli to individual neuronal subpopulations.

Most of these desynchronization techniques were developed for networks with fixed connectivity. In the brain, however, synaptic plasticity leads to dynamic reorganizations of neuronal networks (Doidge, 2007; Liu et al., 2015; Van Ooyen and Butz-Ostendorf, 2017; Eagleman, 2020). One plasticity mechanism is spike-timing-dependent plasticity (STDP), where the change of the strengths of synaptic connections depends on the relative timing of post- and presynaptic spikes (Markram et al., 1997; Abbott and Nelson, 2000; Caporale and Dan, 2008). In many brain regions, STDP leads to a strengthening of synapses, if the postsynaptic neuron fires shortly after the presynaptic neuron, and to a weakening in the opposite case (Markram et al., 1997; Bi and Poo, 1998). STDP may lead to the formation of strongly connected neuronal assemblies (Litwin-Kumar and Doiron, 2014) and may stabilize certain patterns of neuronal activity, e.g., synchronized activity (Karbowski and Ermentrout, 2002). Plasticity can also lead to multistability in neuronal networks and networks of oscillators. In particular, networks with coexisting stable states, such as cluster states, desynchronized states, and synchronized states have been studied (Seliger et al., 2002; Zanette and Mikhailov, 2004; Tass and Majtanik, 2006; Masuda and Kori, 2007; Maistrenko et al., 2007; Aoki and Aoyagi, 2009; Berner et al., 2020; Yanchuk et al., 2020).

Extensive theoretical and computational studies on CR stimulation of multistable plastic networks showed that CR stimulation may reshape the synaptic connectivity, and cause long-lasting desynchronization by driving the network into the attractor of a stable desynchronized state (Tass and Majtanik, 2006; Hauptmann and Tass, 2009; Popovych and Tass, 2012; Lourens et al., 2015; Manos et al., 2018; Kromer and Tass, 2020; Kromer et al., 2020). Corresponding long-lasting therapeutic effects and/or sustained reduction of neuronal synchrony were confirmed in preclinical *in vitro* studies (Tass et al., 2009), in preclinical *in vivo* studies (Tass et al., 2012b; Wang et al., 2016) as well as in clinical studies, delivering CR stimulation through implanted DBS electrodes (Adamchic et al., 2014) or noninvasively by means of vibrotactile CR fingertip stimulation (Tass, 2017; Syrkin-Nikolau et al., 2018; Pfeifer et al., 2021).

Detailed computational studies on CR stimulation of plastic neuronal networks suggested that long-lasting desynchronization effects may be sensitive to the ratio of the stimulation frequency and the dominant frequency of the pathological synchronous rhythm, *f*_rhythm_ (Manos et al., 2018). Presumably, this is because stimuli are delivered with fixed inter-stimulus intervals which may lead to unfavorable resonances with other time scales (Kromer and Tass, 2020). A lack of frequency robustness might restrict clinical application as individual symptoms during PD are related to pathological synchrony in different frequency bands. Specifically, synchronized basal ganglia activity in the theta band (3−10 Hz) has been associated with symptoms such as dyskinesia and tremor (Brown, 2003; Steigerwald et al., 2008; Tass et al., 2010; Contarino et al., 2012), whereas synchronized beta band activity (13−30 Hz) has been associated with symptoms such as rigidity and bradykinesia (Kühn et al., 2006; Weinberger et al., 2006). Furthermore, tremor may be associated with different central oscillators (Raethjen et al., 2000).

In order to increase the frequency robustness of long-lasting effects, spatial and temporal randomization of stimulus deliveries was suggested (Kromer and Tass, 2020). In particular, random reset (RR) stimulation was introduced (Kromer and Tass, 2020) during which stimuli are delivered at random times to randomly selected neuronal subpopulations. During RR stimulation, temporal randomization is realized by choosing stimulation times according to a Poisson spike train. Spatial randomization is achieved by delivering stimuli to randomly selected neuronal subpopulations. However, in Kromer and Tass (2020) no spatial relations between neurons were considered, this was somewhat artificial and could not be directly applied to a DBS-type setup as it implicitly assumed microscopic control, i.e., it assumed that individual neurons could be stimulated independently. Compared to CR stimulation, RR stimulation presents an extreme case with minimal temporal and spatial correlations between stimulus deliveries (Kromer and Tass, 2020; Khaledi-Nasab et al., 2021b). It was found that long-lasting desynchronization effects of RR stimulation were more robust with respect to parameter changes than those of CR stimulation, whereas acute desynchronization effects of RR stimulation were significantly weaker (Kromer and Tass, 2020; Khaledi-Nasab et al., 2021b). Also, temporal randomization during RR stimulation helped to avoid stimulus deliveries during the neurons' refractory periods. The authors reported that the resulting synaptic reshaping was more likely to drive the network into a stable desynchronized state.

It was argued that improved parameter robustness resulted from two effects: First, for CR stimulation with unfavorable stimulation frequencies, a significant portion of stimuli might be delivered during the neurons' refractory periods. This renders such stimulation protocols ineffective. In contrast, temporal variability of stimulus deliveries during RR stimulation improved the robustness of long-lasting effects of RR stimulation relative to CR stimulation. Second, RR stimulation led to a broader distribution of time lags between post- and presynaptic spike times that determined weight updates due to STDP. Thus, a bigger part of the STDP function was considered for weight updates. It was found that while CR with fine-tuned stimulation frequency led to faster weight reduction, weight reduction during RR was more robust with respect to changes of the stimulation frequency (Kromer and Tass, 2020).

In the present paper, we hypothesize that appropriately adding temporal and/or spatial randomness to CR stimulus patterns might improve the parameter robustness of long-lasting CR effects, thereby preserving its pronounced acute desynchronization effects. To this end, we analyze the effect of temporal and spatial correlations in spatio-temporal stimulus patterns on acute and long-lasting desynchronization effects of the stimulation. First, we consider a classic CR stimulation pattern (Tass, 2003a; Kromer et al., 2020). Then, we reduce temporal and spatial correlations between stimulus delivery times. Temporal correlations are reduced by adding a random jitter to the stimulus delivery times. This results in a *noisy* CR pattern (NCR). Spatial correlations are reduced by shuffling the sequence of stimulation site activations. This results in a *shuffled* CR pattern (SCR). Finally, both jitter and shuffling are applied, leading to a *shuffled noisy* CR pattern (SNCR). Employing theoretical analysis and computer simulations of plastic neuronal networks, we analyze and compare the effect of random jitter and spatial shuffling on the long-lasting outcome of stimulation. Our results suggest that random jitter is more suitable for increasing the frequency robustness of long-lasting desynchronization effects than spatial shuffling. Of note, random jitter does not degrade acute desynchronization effects.

This paper is organized as follows: In section 2, we introduce the model and the different stimulation patterns used throughout the manuscript. In section 2.6, we derive theoretical predictions of the stimulation-induced synaptic weight dynamics for different stimulation patterns in the limit of strong and fast stimulation. Then, in section 3, we compare theoretical predictions to results from numerical simulations of networks of leaky integrate-and-fire (LIF) neurons with STDP. Furthermore, we present results for weak stimulation. We find that random jitter is more suitable for increasing parameter robustness of long-lasting effects than shuffling. Finally, in section 4 we provide a detailed discussion of our results.

## 2. Model and Methods

### 2.1. Neuronal Network Model

Throughout the paper, we consider networks of 10^3^ excitatory LIF neurons with STDP (Kromer et al., 2020; Khaledi-Nasab et al., 2021b). Parameters are chosen according to Kromer et al. (2020) and Khaledi-Nasab et al. (2021b) such that a stable synchronized and a stable desynchronized states coexist. See Appendix for more details. Neurons are equidistantly spaced in the interval *x*_*i*_ ∈ [−2.5, 2.5] mm. This is motivated by the length of the short axes of an elipsoidal volume approximation of the human STN used in a detailed computational study (Ebert et al., 2014). Each neuron has *N*_syn_ = 0.07*N* outgoing synapses, where the probability for a synaptic connection between neurons *i* and *j* is proportional to ∝ exp((|*x*_*j*_ − *x*_*i*_|)/0.5 mm) (Ebert et al., 2014). Throughout the paper, simulation results are averaged over three different network realizations, i.e., different realizations of random network connectivity.

Initially, synaptic weights *w*_*ij*_(*t* = 0) are randomly set to either one or zero, such that a mean synaptic weight of 〈*w*(*t* = 0)〉 = 0.5 is realized. Here, *i* and *j* refer to the pre- and postsynaptic neurons, respectively. Each network realization was simulated until it approached a stable synchronized state, see Kromer et al. (2020) and Khaledi-Nasab et al. (2021b).

Stimulation is applied to *N*_s_ stimulation sites. To this end, we divide the interval of possible *x* coordinates into *N*_s_ equal segments. Neurons with coordinates in the *k*th segment are assumed to receive stimuli delivered to the *k*th stimulation site. Throughout the paper, we will refer to these neurons as the *k*th subpopulation. Neurons in the same subpopulation receive stimuli simultaneously and with equal strength. We thereby neglect distance-dependent modulations of the received stimulation current and finite travel times of the electrical signal through the tissue.

### 2.2. Spike-Timing Dependent Plasticity

The dynamics of synaptic weights *w*_*ij*_(*t*) is determined by STDP. We consider a nearest-neighbor STDP scheme in which weight updates are performed at postsynaptic spike times and presynaptic spike arrival times (Morrison et al., 2008). Corresponding weight updates *w*_*ij*_ → *w*_*ij*_ + *W*(*t*_*j*_ − (*t*_*i*_ + *t*_d_)) are given by the STDP function (Kromer and Tass, 2020; Song et al., 2000)


(1) $$W(\Delta t)=\eta \left\{ \begin{array}{l} e^{-|\Delta t|/\tau_{+}},\;\Delta t>0\\ 0.\;\Delta t=0.\\ -\frac{\beta }{\tau_{R}}\;e^{-|\Delta t|/\tau_{-}},\;\Delta t<0 \end{array}\right.$$

Here, Δ*t* = *t*_*j*_ − (*t*_*i*_ + *t*_d_) is the time lag between the current postsynaptic spike time *t*_*j*_ and the latest presynaptic spike arrival time *t*_*i*_ + *t*_d_ (if the update is done at a postsynaptic spike time), or the time lag between the current presynaptic spike arrival time *t*_*i*_ + *t*_d_ and the latest postsynaptic spike time *t*_*j*_ (if the update is performed at a presynaptic spike arrival time). η = 0.02 scales the weight update per spike, τ_*R*_ = 4 yields asymmetry in STDP decay times, τ_+_ = 10 ms and τ_−_ = τ_+_ τ_*R*_, β = 1.4 scales the ratio of overall long-term depression (LTD) to long-term potentiation (LTP).

These STDP parameters lead to the coexistence of a strongly connected state with synchronized neuronal activity and a weakly connected state with asynchronous neuronal activity (Kromer and Tass, 2020; Kromer et al., 2020; Khaledi-Nasab et al., 2021a,b).

### 2.3. Stimulation Patterns

In the present paper, we consider four stimulation patterns: a (regular) CR pattern and three randomized CR patterns. Randomization is obtained by adding random jitters to the stimulation times (reduced temporal correlations between stimuli), shuffling of the sequence of stimulation sites (reduced spatial correlations between stimuli), and a combination of both random jitters and shuffling (reduced temporal and spatial correlations between stimuli). In the following, we introduce these four stimulation patterns in detail.

- **Regular CR stimulation (CR):** CR stimulation is delivered in cycles of *N*_s_ stimuli (Tass, 2003b), where *N*_s_ is the number of stimulation sites. During each CR cycle, each site receives exactly one stimulus, and stimuli are administered at times $t_{0}+\frac{\left(k+0.5\right)T_{\text{CR}}}{N_{\text{s}}}$, *k* = 0, 1, .., *N*_s_ − 1. Here *t*_0_ is the beginning of the CR cycle and *T*_CR_ = 1/*f*_CR_ is the cycle length. *f*_CR_ is the stimulation frequency and corresponds to the mean frequency at which individual sites receive stimuli.
  In several preclinical and clinical studies (Tass et al., 2012b; Adamchic et al., 2014; Pfeifer et al., 2021), the sequence of stimulation site activations during each cycle was chosen at random for each cycle. The resulting version of CR stimulation is referred to as CR with rapidly varying sequence in the literature (Popovych and Tass, 2012; Zeitler and Tass, 2015). For the sake of brevity, we will refer to this pattern as CR.
- **Noisy CR stimulation with random jitters (NCR):** Same as CR stimulation except that there are random jitters added to the stimulation times *s*_*k*_. Jitters are uniformly distributed in the interval $s_{k}\in \left[-\sigma_{\text{CR}}\frac{T_{\text{CR}}}{2N_{\text{s}}},\sigma_{\text{CR}}\frac{T_{\text{CR}}}{2N_{\text{s}}}\right)$ with 0 ≤ σ_CR_ ≤ 1. Thus, stimuli during a CR cycle are delivered at random times $t_{0}+\frac{\left(k+0.5\right)T_{\text{CR}}}{N_{\text{s}}}+s_{k}$, *k* = 0, 1, .., *N*_s_ − 1. The case σ_CR_ = 1, refers to the limit of maximum variability of stimulus onset times. We exclude larger values of σ_CR_ as these would result in overlapping intervals of possible stimulus onset times.
  A corresponding stimulation pattern with moderate jitters was used in a clinical study on vibrotactile CR stimulation of Parkinson's patients (Pfeifer et al., 2021). There, it was referred to as noisy CR (NCR). We will refer to CR with random jitter as NCR stimulation.
- **Shuffled CR stimulation (SCR)**: Same as CR stimulation except that stimuli are delivered to randomly selected sites. Thus, the restriction that each site receives exactly one stimulus per cycle was lifted. Sites are selected for stimulus delivery with uniform probability.
  This stimulation pattern was used in a previous study on stimulation-induced desynchronization (Tass and Hauptmann, 2009). There, it was referred to as multi-site random-site stimulation (Tass and Hauptmann, 2009). We will refer to shuffled CR stimulation as SCR stimulation.
- **Shuffled Noisy CR with random jitters (SNCR):** Same as SCR but with random jitters added to the stimulus delivery times. We will refer to shuffled CR with random jitters as SNCR stimulation. In the limit of small jitter, σ_CR_ → 0, SNCR stimulation becomes equivalent to SCR stimulation.

For NCR and SNCR stimulation, the parameter σ_CR_ scales the width of the distribution for random jitters and therefore the reduction of temporal correlations between stimulus delivery times. In the limit of σ_CR_ = 0, the NCR pattern is equivalent to the CR pattern, and the SNCR pattern is equivalent to the SCR pattern.

Individual stimuli are charge-balanced and consist of excitatory and inhibitory pulses with durations of ν_e_ = 0.5 ms and ν_i_ = 3 ms, respectively. This asymmetry is motivated by preclinical and clinical studies on CR stimulation employing asymmetric pulse shapes (Wang et al., 2016; Adamchic et al., 2014). The excitatory pulse has the amplitude $A_{\text{e}}=A_{\text{stim}}\mu /\nu_{\text{e}}$ and the inhibitory pulse the amplitude $A_{\text{i}}=-A_{\text{stim}}\mu /\nu_{\text{i}}$. The two pulses are separated by a gap of 0.2 ms. Here, μ = (*V*_th,spike_ − *V*_reset_)/〈*C*_*i*_〉 and *A*_stim_ is the stimulation strength. (*V*_th,spike_ − *V*_reset_) is the voltage difference between the maximum spiking threshold *V*_th,spike_ and the voltage reset *V*_reset_. 〈*C*_*i*_〉 is the mean membrane capacitance. See Appendix for more details and see Pyragas et al. (2018) for a discussion of optimal waveforms for DBS.

**Figure 1:**
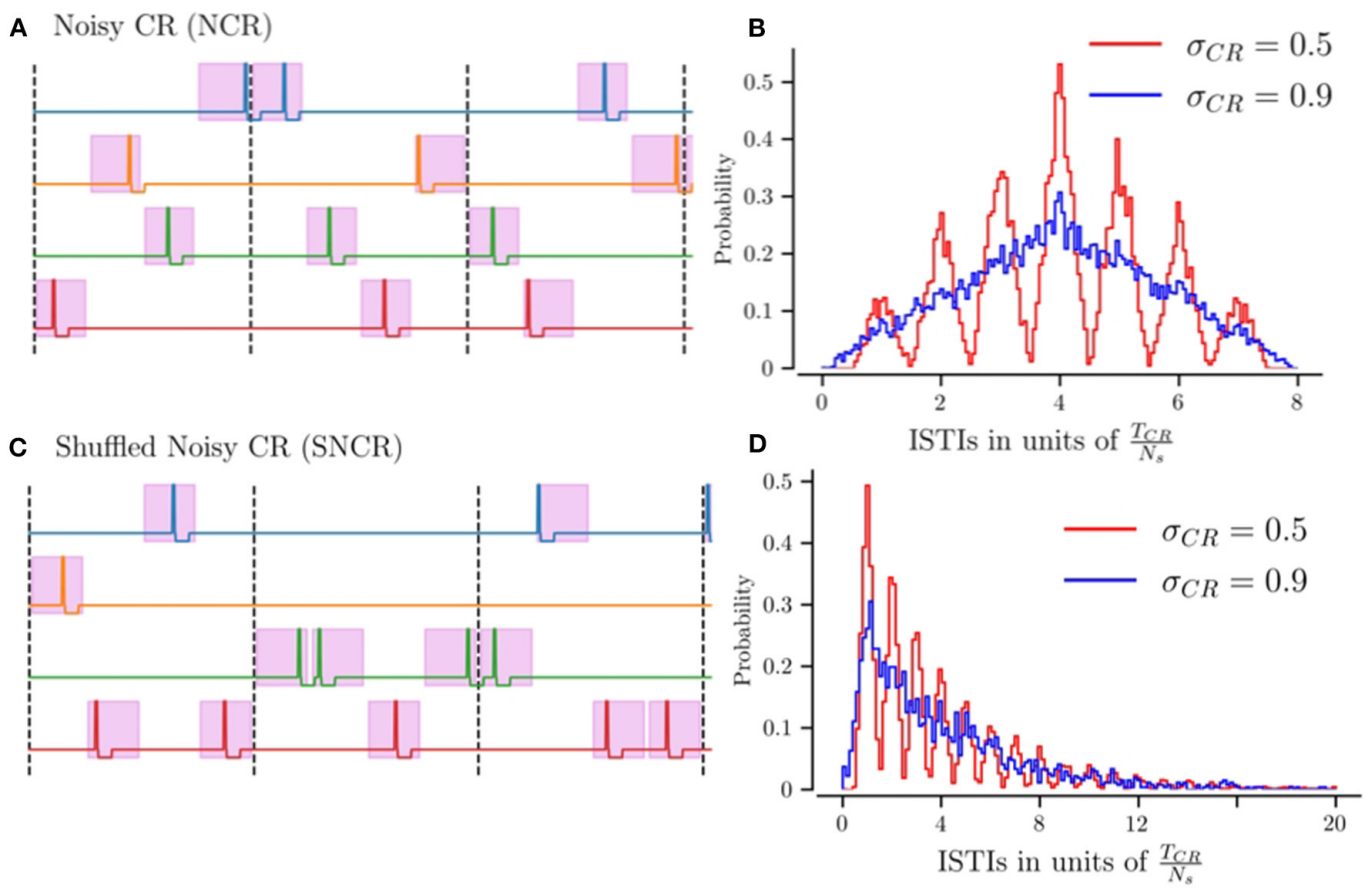
Illustration of stimulation patterns used throughout the manuscript and the resulting distribution of inter-stimulus intervals. **(A)** Possible realization of NCR stimulation for *N*_s_ = 4 stimulation sites. Colored curves indicate the stimulation currents delivered to the individual sites. The pink region marks intervals of possible stimulus onset times, for the maximum jitter σ_CR_ = 1. The limit of vanishing jitter, σ_CR_ = 0, corresponds to deterministic stimulus onsets, i.e., CR stimulation. Vertical dashed lines separate subsequent CR cycles, with cycle period *T*_CR_ = 1/*f*_CR_. **(B)** The distribution of inter-stimulus intervals (ISTIs) for NCR stimulation for two values of stimulus jitter, σ_CR_. **(C)** Possible realization of SNCR stimulation with *N*_s_ = 4 stimulation sites. The color code is the same as in **(A)**. **(D)** The distribution of ISTIs for SNCR stimulation for two values of σ_CR_.

Figures 1A,C shows realizations of stimulus patterns for NCR and SNCR. The shaded areas mark possible stimulus onset times. Figures 1B,D shows the distribution of inter-stimulus intervals (ISTIs) for two values of σ_CR_. Small values of σ_CR_ lead to a narrow distribution with peaks at integer multiples of *T*_CR_/*N*_s_, while larger σ_CR_ lead to broader ISTI distributions.

NCR with minimal temporal correlations, σ_CR_ = 1, leads to ISTIs between zero and 2*T*_CR_ with mean ISTI *T*_CR_. For SNCR, the ISTI distribution attains its global maximum at *T*_CR_/*N*_s_ and decreases for larger ISTIs. For large jitters, σ_CR_ ≈ 1 it decays approximately exponentially, see Figure 1D. Consequently, individual sites may not receive any stimulus for multiple cycles.

### 2.4. Quantification of Synchronization

In order to quantify the degree of neuronal synchrony, we calculate the time-averaged Kuramoto order parameter (Kuramoto, 1984)


(2) $$\begin{array}{l} \bar{\rho }\left(t\right)=\frac{1}{t_{k}}\overset{t+\frac{t_{k}}{2}}{\underset{t-\frac{t_{k}}{2}}{\int }}dt^{\prime }(\frac{1}{N}\overset{N-1}{\underset{k=0}{\sum }}e^{2\pi I\psi_{k}\left(t^{\prime }\right)}). \end{array}$$

Here, *t*_*k*_ = 10 s is an averaging interval and *N* is the number of neurons. ψ_*k*_(*t*) is a phase function associated with the spiking of neuron *k*. ψ_*k*_(*t*) attains subsequent integer values at subsequent spike times and increases linearly during interspike intervals (Rosenblum et al., 2001). $\bar{\rho }\approx 1$ indicates pronounced in-phase synchronization, whereas the absence of in-phase synchronized neuronal activity leads to $\bar{\rho }\approx 0$.

### 2.5. Data Evaluation

In simulations, the data for the mean synaptic weight, 〈*w*〉, and the time-averaged Kuramoto order parameter, $\bar{\rho }$, were recorded every 10 s. Acute effects of stimulation are quantified using the acute mean synaptic weight, 〈*w*〉_ac_, which is the mean weight at the end of the stimulation period. For sufficiently low 〈*w*〉_ac_ the network approaches a stable desynchronized state after cessation of stimulation (see Kromer et al., 2020; Khaledi-Nasab et al., 2021b).

In order to quantify the degree of acute synchronization, we time-averaged the Kuramoto order parameter, ${\bar{\rho }}_{\text{ac}}$, over the last 10 s of the stimulation duration. Accordingly, we quantified the acute after-effect of stimulation by time-averaging the Kuramoto order parameter, ${\bar{\rho }}_{\text{af}}$, over the first 10 s interval after cessation of the stimulation. Lastly, long-lasting effects of stimulation are quantified by means of the time-averaged Kuramoto order parameter, ${\bar{\rho }}_{\text{ll}}$, which is evaluated over a 10 s interval 1,000 s after the stimulation ceases. ${\bar{\rho }}_{\text{ll}}\approx 0$ indicates long-lasting desynchronization, while ${\bar{\rho }}_{\text{ll}}\approx 1$ indicates that stimulation did not entail long-lasting desynchronization effects.

### 2.6. Estimated Weight Change During Strong and Fast Stimulation

We derive estimates for the stimulation-induced weight dynamics during stimulation by applying the theoretical framework presented in Kromer and Tass (2020). In the following, we present the main steps and derive specific results for NCR, SCR, and SNCR. Results for CR were previously reported in Kromer et al. (2020) and are given below as a reference for the reader's convenience.

We consider a synapse with synaptic weight *w*_*ij*_(*t*), presynaptic neuron *i*, and postsynaptic neuron *j*. Its mean rate of weight change during a time interval of duration *T*, starting at time *t*, is given by Kempter et al. (1999)


(3) $$\begin{array}{l} J_{ij}\left(t,T\right):=\frac{w_{ij}\left(t+T\right)-w_{ij}\left(t\right)}{T}\\ \;=\frac{1}{T}\underset{t_{i},t_{j}\;\in \text{spike pairs}}{\sum }W\left(t_{j}-\left(t_{i}+t_{d}\right)\right). \end{array}$$

*W*(*t*) is the STDP function given in Equation (1). *t*_*i*_ and *t*_*j*_ are the pre- and postsynaptic spike times, respectively. The sum runs over all pairs of pre- and postsynaptic spike times, that contribute to weight changes according to the given STDP scheme (Morrison et al., 2008). In the present paper, we consider a nearest-neighbor scheme in which each presynaptic spike arrival time (*t*_*i*_ + *t*_d_) is paired with the latest postsynaptic spike time (*t*_*j*_) and each postsynaptic spike time is paired with the latest presynaptic spike arrival time.

We are particularly interested in the expectation value $\langle J_{ij}\left(t,T\right)\rangle$, which is obtained by averaging over different realizations of the stimulation pattern. Assuming stationary dynamics and the time interval *T* being long compared to the interspike intervals as well as the characteristic time scale of temporal correlations in the stimulation pattern, the mean rate of weight change becomes independent of the starting point *t* and the length *T* of the time interval. Then, we can approximate $\langle J_{ij}\left(t,T\right)\rangle$ by its limit for long time intervals $\langle J_{ij}\left(t,T\right)\rangle \to \langle J_{ij}^{\infty }\rangle$.

Next, we restrict ourselves to the case of stimulation-controlled spiking where each spike is caused by a stimulus and each stimulus causes a spike of the stimulated neurons. In simulations of the LIF network, this can be realized for weak synaptic interaction by considering strong stimulation (*A*_stim_ ≈ 1) that is fast compared to neuronal firing rates in the absence of stimulation. Furthermore, the duration of inhibitory pulses ν_i_ should be short, such that the membrane potential recovers from inhibition before the next stimulus arrives. In the case of stimulation-controlled spiking, the spike times of pre- and postsynaptic neurons can be related to stimulus delivery times by introducing the distribution of spike response times λ(ϵ), where ϵ is the time lag between stimulus delivery and triggered neuronal spiking response (Kromer and Tass, 2020). If both post- and presynaptic neurons receive stimuli at rate *f*_CR_, these assumptions allow us to rewrite Equation (3) as


(4) $$\begin{array}{l} \langle J_{ij}^{\infty }\rangle =f_{\text{CR}}\int dt^{\prime }\;G_{ij}\left(t^{\prime }\right)W\left(t^{\prime }-t_{d}\right). \end{array}$$

$G_{ij}\left(t^{\prime }\right)$ is the distribution of time lags $t^{\prime }=t_{j}-t_{i}$ between pairs of post- and presynaptic spike times that contribute to weight changes.

We calculate $G_{ij}\left(t^{\prime }\right)$ for CR, NCR, SCR, and SNCR. Following, $G_{ij}^{\text{A}}\left(t^{\prime }\right)$ with A=CR, NCR, SCR, or SNCR will denote the distribution of time lags during ongoing stimulation with the respective stimulation protocol. To this end, we consider the statistics of time lags *t*′ between subsequent post- and presynaptic spikes. We set *t*′ = *S* + Δ with the inter-stimulus interval *S* between stimuli triggering these spikes and Δ = ϵ_post_ − ϵ_pre_ denoting the time difference between the realizations of spike response times for the considered spikes of the post- and presynaptic neurons resulting in the time lag *t*′. For narrow distributions of spike response times λ(*t*), $G_{ij}^{\text{A}}\left(t^{\prime }\right)$ in Equation (4) can be approximated using the two distributions: $\Lambda \left(t\right)=\int_{-\infty }^{\infty }dt^{\prime \prime }\;\lambda \left(t^{\prime \prime }\right)\lambda \left(t^{\prime \prime }+t\right)$ and $p_{ij}^{\text{A}}\left(S|\Delta \right)$ as


(5) $$\begin{array}{l} G_{ij}^{\text{A}}\left(t\right)\approx \int d\Delta \;\Lambda \left(\Delta \right)\int dS\;\delta \left(t-S-\Delta \right)p^{\text{A}}\left(S|\Delta \right), \end{array}$$

see also Kromer and Tass (2020) and Kromer et al. (2020). $p_{ij}^{\text{A}}\left(S|\Delta \right)$ is the distribution of inter-stimulus intervals between stimuli delivered to the post- and presynaptic neurons for which the resulting pairs of spike times contribute to weight changes according to the STDP scheme. Note that $G_{ij}^{\text{A}}\left(t\right)$ and *p*^A^(*S*|Δ) are in general not normalized to one as multiple intervals per spike can contribute to weight changes. For the STDP scheme considered in the present paper, both are normalized to two, i.e., $\int_{-\infty }^{\infty }dt\;G_{ij}^{\text{A}}\left(t\right)=2$ and $\int_{-\infty }^{\infty }dS\;p^{\text{A}}\left(S|\Delta \right)=2$.

Estimates $\langle J_{ij}^{\infty }\rangle$ of the mean rate of weight change for the four different stimulation patterns considered in the present paper can be obtained by calculating $p_{ij}^{\text{A}}\left(S|\Delta \right)$. The latter depends on the conditional probability for the postsynaptic neuron to receive a stimulus at time *S* after a stimulus has been delivered to the presynaptic neuron and vice versa.

For the stimulus patterns considered in the present paper, two classes of synapses exist, each characterized by a distinct statistics of stimulus delivery times to the post- and presynaptic neurons, see Kromer and Tass (2020). *Intrapopulation* synapses connect neurons that are affected by the same stimulation site. Hence, post- and presynaptic neurons receive stimuli simultaneously. In contrast, *interpopulation* synapses connect neurons that are affected by different stimulation sites. Thus, post- and presynaptic neurons receive stimuli at different times. Following, we will replace the indices *i* and *j*, referring to the connected neurons, by the terms ‘intra' and ‘inter' referring to the respective type of synapse. Specifically, we set $p_{ij}^{\text{A}}\left(S|\Delta \right)\to p_{\text{intra}}^{\text{A}}\left(S|\Delta \right)$, if neurons *i* and *j* are in the same subpopulation, and $p_{ij}^{\text{A}}\left(S|\Delta \right)\to p_{\text{inter}}^{\text{A}}\left(S|\Delta \right)$, if neurons *i* and *j* are in different subpopulations.

Results for CR stimulation have been derived in Kromer and Tass (2020) and Kromer et al. (2020) and will be presented below for the reader's convenience. In the present paper, we consider the limit of sharp distributions of spike response times λ(ϵ) = δ(ϵ), thereby focusing on the contribution of the distribution of inter-stimulus intervals to weight changes. In this limit, we derive $p_{\text{intra}}^{\text{A}}\left(S\right)=p_{\text{intra}}^{\text{A}}\left(S|\Delta =0\right)$ and $p_{\text{inter}}^{\text{A}}\left(S\right)=p_{\text{inter}}^{\text{A}}\left(S|\Delta =0\right)$ for A = NCR, SCR, and SNCR stimulation.

#### 2.6.1. CR and NCR Stimulation

We calculate the distributions of inter-stimulus intervals that contribute to weight changes for CR and NCR stimulation $p_{\text{intra/inter}}^{\text{CR}}\left(S\right)$ and $p_{\text{intra/inter}}^{\text{NCR}}\left(S\right)$, respectively.

In Kromer and Tass (2020) and Kromer et al. (2020), $p_{\text{intra/inter}}^{\text{CR}}\left(S\right)$ has been derived for the case of *N*_s_ = 4 and arbitrary *N*_s_, respectively. Note that *N*_s_ was called *M* in the cited papers. Furthermore, in Kromer et al. (2020) a nonlinear dependence of $p_{\text{inter}}^{\text{CR}}\left(S|\Delta \right)$ on stimulation parameters was found. In more detail, if *S* + Δ < *t*_d_ presynaptic spikes arrive at the postsynaptic neuron after the next spikes have been initiated by stimuli. In order to account for this effect, a correction term was suggested. In the case of Λ(Δ) = δ(Δ) the authors' approach yields


(6) $$\begin{array}{l} p_{\text{intra/inter}}^{\text{CR}}(S)=\{\begin{array}{ll} p_{\text{intra/inter},0}^{\text{CR}}(S), & \frac{1}{N_{\text{S}}f_{\text{CR}}}\geq t_{\text{d}}\\ p_{\text{intra/inter},0}^{\text{CR}}(S)+\delta p_{\text{intra/inter}}^{\text{CR}}(S), & \frac{1}{N_{\text{S}}f_{\text{CR}}}<t_{\text{d}} \end{array}. \end{array}$$

Here, $p_{\text{intra/inter},0}^{\text{CR}}\left(S\right)$ and $\delta p_{\text{intra/inter}}^{\text{CR}}\left(S\right)$ are the zeroth order term ($\frac{1}{N_{\text{S}}f_{\text{CR}}}\geq t_{\text{d}}$) and the first correction term accounting for the case that presynaptic spikes arrive at postsynaptic neurons after the next but before the second to next stimulus has been delivered, i.e., inter-stimulus intervals are smaller then the delay time, but second order inter-stimulus intervals are larger than the delay time. In the present paper, we neglect further correction terms. These would account for more stimulus deliveries during a single delay time *t*_d_.

The results of Kromer and Tass (2020) and Kromer et al. (2020) can be expanded to NCR by considering that individual stimulation times are uniformly distributed around their mean. Consequently, while $p_{\text{intra},0}^{\text{CR}}$ is given by a superposition of delta distributions at multiples of $\frac{1}{N_{\text{s}}f_{\text{CR}}}$, $p_{\text{intra},0}^{\text{NCR}}$ shows additional variability which can be described by the distribution *q*_σ_CR__(*S*) that is centered at zero. For a given width, σ_CR_, of the distribution of jitters, we find


(7) $$\begin{array}{l} p_{\text{intra},0}^{\text{CR/NCR}}\left(S\right)=\delta \left(S\right)+\overset{2N_{\text{s}}}{\underset{m=1}{\sum }}\frac{N_{\text{s}}-|m-N_{\text{s}}|}{N_{\text{s}}^{2}}q_{\sigma_{\text{CR}}}\left(S-\frac{m}{N_{\text{s}}f_{\text{CR}}}\right), \end{array}$$

with


(8) $$\begin{array}{l} q_{\sigma_{\text{CR}}}(S)=\frac{N_{\text{s}}^{2}f_{\text{CR}}^{2}}{\sigma_{\text{CR}}^{2}}\{\begin{array}{ll} -|S|+\frac{\sigma_{\text{CR}}}{f_{\text{CR}}N_{\text{s}}}, & -\frac{\sigma_{\text{CR}}}{f_{\text{CR}}N_{\text{s}}}<S<\frac{\sigma_{\text{CR}}}{f_{\text{CR}}N_{\text{s}}}\\ 0, & \text{otherwise} \end{array},\;\sigma_{\text{CR}}>0. \end{array}$$

Equation (8) is the distribution of the difference between two random variables that are uniformly distributed in the interval [−σ_CR_/(2*N*_s_*f*_CR_), σ_CR_/(2*N*_s_*f*_CR_)]. These two random variables correspond to the jitters of post- and presynaptic stimulus delivery times. The case of CR stimulation (σ_CR_ = 0) can be described by *q*_0_(*S*): = δ(*S*), which yields the results of Kromer and Tass (2020).

Accordingly, we derive the first correction term using the results for CR stimulation published in Kromer et al. (2020)


(9) $$\begin{array}{l} \delta p_{\text{intra}}^{\text{CR/NCR}}\left(S\right)=-\frac{1}{N_{\text{s}}^{2}}\delta \left(S\right)+\overset{N_{\text{s}}-1}{\underset{\xi =0}{\sum }}\frac{q_{\sigma_{\text{CR}}}\left(S-\frac{N_{\text{s}}+1+\xi }{f_{\text{CR}}N_{\text{s}}}\right)}{N_{\text{s}}^{3}},\;\frac{1}{N_{\text{s}}f_{\text{CR}}}<t_{\text{d}}. \end{array}$$

We apply the same approach to the results for interpopulation synapses. Based on the results for CR stimulation from Kromer et al. (2020), we obtain


(10) $$\begin{array}{l} p_{\text{inter},0}^{\text{CR/NCR}}(S)=\sum_{\xi =1}^{N_{\text{s}}}[\frac{1}{N_{\text{s}}^{2}}\left(1+\sum_{k=2}^{\xi }\left(1-\frac{k-1}{N_{\text{s}}-1}\right)\right)\\ +\frac{N_{\text{s}}-\xi }{N_{\text{s}}(N_{\text{s}}-1)}]\times [q_{\sigma_{\text{CR}}}\left(S+\frac{\xi }{N_{\text{s}}f_{\text{CR}}}\right)\\ +q_{\sigma_{\text{CR}}}\left(S-\frac{\xi }{N_{\text{s}}f_{\text{CR}}}\right)]+\overset{2N_{\text{s}}-2}{\underset{\xi =N_{\text{s}}+1}{\sum }}\left[\frac{1}{N_{\text{s}}^{2}}\sum_{k=\xi -N_{\text{s}}}^{N_{\text{s}}-2}\left(1-\frac{k}{N_{\text{s}}-1}\right)\right]\\ \times \left(q_{\sigma_{\text{CR}}}\left(S+\frac{\xi }{N_{\text{s}}f_{\text{CR}}}\right)+q_{\sigma_{\text{CR}}}\left(S-\frac{\xi }{N_{\text{s}}f_{\text{CR}}}\right)\right). \end{array}$$

For the first correction term, we find


(11) $$\begin{array}{l} \delta p_{\text{inter}}^{\text{CR/NCR}}\left(S\right)=\overset{N_{\text{s}}-2}{\underset{u=0}{\sum }}\overset{N_{\text{s}}-1}{\underset{v=0}{\sum }}\frac{q_{\sigma_{\text{CR}}}(S-\frac{B_{uv}}{f_{\text{CR}}N_{\text{s}}})-q_{\sigma_{\text{CR}}}(S+\frac{C_{uv}}{f_{\text{CR}}N_{\text{s}}})}{N_{\text{s}}^{2}\left(N_{\text{s}}-1\right)}\\ +\overset{N_{\text{s}}-1}{\underset{v=0}{\sum }}\frac{q_{\sigma_{\text{CR}}}(S-\frac{D_{uv}}{f_{\text{CR}}N_{\text{s}}})}{N_{\text{s}}^{3}}-\overset{N_{\text{s}}-1}{\underset{v=1}{\sum }}\frac{q_{\sigma_{\text{CR}}}(S+\frac{v}{f_{\text{CR}}N_{\text{s}}})}{N_{\text{s}}^{2}\left(N_{\text{s}}-1\right)},\;\frac{1}{N_{\text{s}}f_{\text{CR}}}<t_{\text{d}}. \end{array}$$

Here, we introduced *B*_*uv*_ = *N*_s_ − *u* + *v*, *C*_*uv*_ = 1 + *u* + *v*, and *D*_*uv*_ = *M* + *u* + *v*.

#### 2.6.2. SCR and SNCR Stimulation

Next, we consider SCR and SNCR stimulation. As stimuli are delivered to random stimulation sites independently of previous stimulus deliveries, the derivation of $p_{\text{intra},0}^{\text{SCR}}\left(s\right)$ and $p_{\text{inter},0}^{\text{SCR}}\left(s\right)$ is comparable to that of the results for RR stimulation presented in Khaledi-Nasab et al. (2021a). Specifically, $p_{\text{intra},0}^{\text{SCR}}\left(S\right)$ results from


(12) $$\begin{array}{l} p_{\text{intra},0}^{\text{SCR/SNCR}}\left(S\right)=\delta \left(S\right)+F\left(S,\sigma_{\text{CR}}\right), \end{array}$$

with the series


(13) $$\begin{array}{l} F\left(S,\sigma_{\text{CR}}\right)=\overset{\infty }{\underset{k=1}{\sum }}\frac{1}{N_{\text{s}}}{\left(1-\frac{1}{N_{\text{s}}}\right)}^{k-1}q_{\sigma_{\text{CR}}}\left(S-\frac{k}{N_{\text{s}}f_{\text{CR}}}\right). \end{array}$$

The summands contain the probabilities 1/*N*_s_ and ${\left(1-1/N_{\text{s}}\right)}^{k-1}$ that the neurons' subpopulations receive the *k*th but not the previous *k* − 1 stimuli.

For interpopulation synapses, we find


(14) $$\begin{array}{l} p_{\text{inter},0}^{\text{SCR/SNCR}}\left(S\right)=F\left(S,\sigma_{\text{CR}}\right)+F\left(-S,\sigma_{\text{CR}}\right). \end{array}$$

The first correction terms $\delta p_{\text{intra}}^{\text{CR/NCR}}\left(S\right)$ and $\delta p_{\text{inter}}^{\text{CR/NCR}}\left(S\right)$ can be derived by considering that inter-stimulus intervals of length $\frac{1}{N_{\text{s}}f_{\text{CR}}}$ result in negative time lags for $t_{d}>\frac{1}{N_{\text{s}}f_{\text{CR}}}$. First, we derive the correction term for intrapopulation synapse. Both post- and presynaptic neurons receive a stimulus at time *t* = 0. If the neurons do not receive a stimulus at time $t=\frac{1}{N_{\text{s}}f_{\text{CR}}}$, the presynaptic spike arrival time 0+*t*_*d*_ is paired with the postsynaptic spike at time *t* = 0 for a negative time lag. However, with probability 1/*N*_s_ both neurons receive a stimulus at time $t=\frac{1}{N_{\text{s}}f_{\text{CR}}}$. Then, the presynaptic spike arrival time *t*_*d*_ is paired with the postsynaptic spike at time $t=\frac{1}{N_{\text{s}}f_{\text{CR}}}$ for a negative time lag. Proceeding accordingly for positive time lags, we find the correction term for intrapopulation synapses


(15) $$\begin{array}{l} \delta p_{\text{intra}}^{\text{CR/NCR}}\left(S\right)=-\frac{1}{N_{\text{s}}}\delta \left(S\right)\\ \;+\frac{1}{N_{\text{s}}^{2}}\overset{\infty }{\underset{k=0}{\sum }}{\left(1-\frac{1}{N_{\text{s}}}\right)}^{k}q_{\sigma_{\text{CR}}}\left(S-\frac{k+2}{N_{\text{s}}f_{\text{CR}}}\right); \end{array}$$

and the following correction terms for interpopulation synapses


(16) $$\begin{array}{l} \delta p_{\text{inter}}^{\text{CR/NCR}}(S)=\frac{1}{N^{2}}\sum_{k=0}^{\infty }{\left(1-\frac{1}{N_{\text{s}}}\right)}^{k}(q_{\sigma_{\text{CR}}}\left(S-\frac{k+2}{N_{\text{s}}f_{\text{CR}}}\right)\\ \;-q_{\sigma_{\text{CR}}}\left(S+\frac{k+1}{N_{\text{s}}f_{\text{CR}}}\right)). \end{array}$$

The first correction term $\delta p_{\text{X}}^{\text{A}}\left(s\right)$, with A = CR, NCR, SCR, or SNCR and X = “intra” or “inter,” needs to be added if postsynaptic spikes, triggered by the next stimulus, occur before the presynaptic spike, triggered by the current stimulus, arrives at the postsynaptic neuron. This scenario changes the order of postsynaptic spike times and presynaptic spike arrival times and occurs with probability $P_{\text{Cor},\sigma_{\text{CR}}}=\int_{0}^{t_{\text{d}}}dS\;q_{\sigma_{\text{CR}}}\left(S-\frac{1}{N_{\text{s}}f_{\text{CR}}}\right)$. By considering the probability for this scenario, we can generalize Equation (6) to the case of NCR and SNCR stimulation as


(17) $$\begin{array}{l} p_{\text{X}}^{\text{A}}\left(S\right)\approx p_{\text{X},0}^{\text{A}}\left(S\right)+P_{\text{Cor},\sigma_{\text{CR}}}\delta p_{\text{X}}^{\text{A}}\left(S\right), \end{array}$$

with σ_CR_ = 0 for CR and SCR stimulation. Using $p_{\text{X}}^{\text{A}}\left(s\right)$ in Equation (5) yields an estimate of $G_{X}^{\text{A}}\left(t\right)$, which can be used in Equation (4) to estimate the mean rate of weight change for stimulation protocol A and synapses of type X.

## 3. Results

We compare long-lasting effects of CR, NCR, SCR, and SNCRstimulation patterns using theoretical estimates of the stimulation-induced weight dynamics and simulations of networks of 10^3^ excitatory LIF neurons with STDP.

In simulations, the network connectivity and the neurons' membrane time capacitances were initialized at random. The networks were prepared in a state of pronounced in-phase synchronization (see section 2). For each network realization, we study the acute, acute after-, and long-lasting effects of stimulation by means of the mean synaptic weight 〈*w*〉 and the Kuramoto order parameter $\bar{\rho }$, Equation (2). Stimulation was delivered for either 500 or 1,000 s, see Figure captions.

### 3.1. Theoretical Predictions for Stimulation-Induced Weight Dynamics

To quantify the stimulation-induced synaptic weight dynamics, we consider the mean rate of weight change during ongoing stimulation. Theoretical estimates, $\langle J_{\text{X}}^{\infty }\rangle$, of the latter have been derived in the Methods section (see Equation 4). We compare the NCR/SNCR stimulation with maximum jitter, σ_CR_ = 1 to the ones without any jitter, CR/SCR.

**Figure 2:**
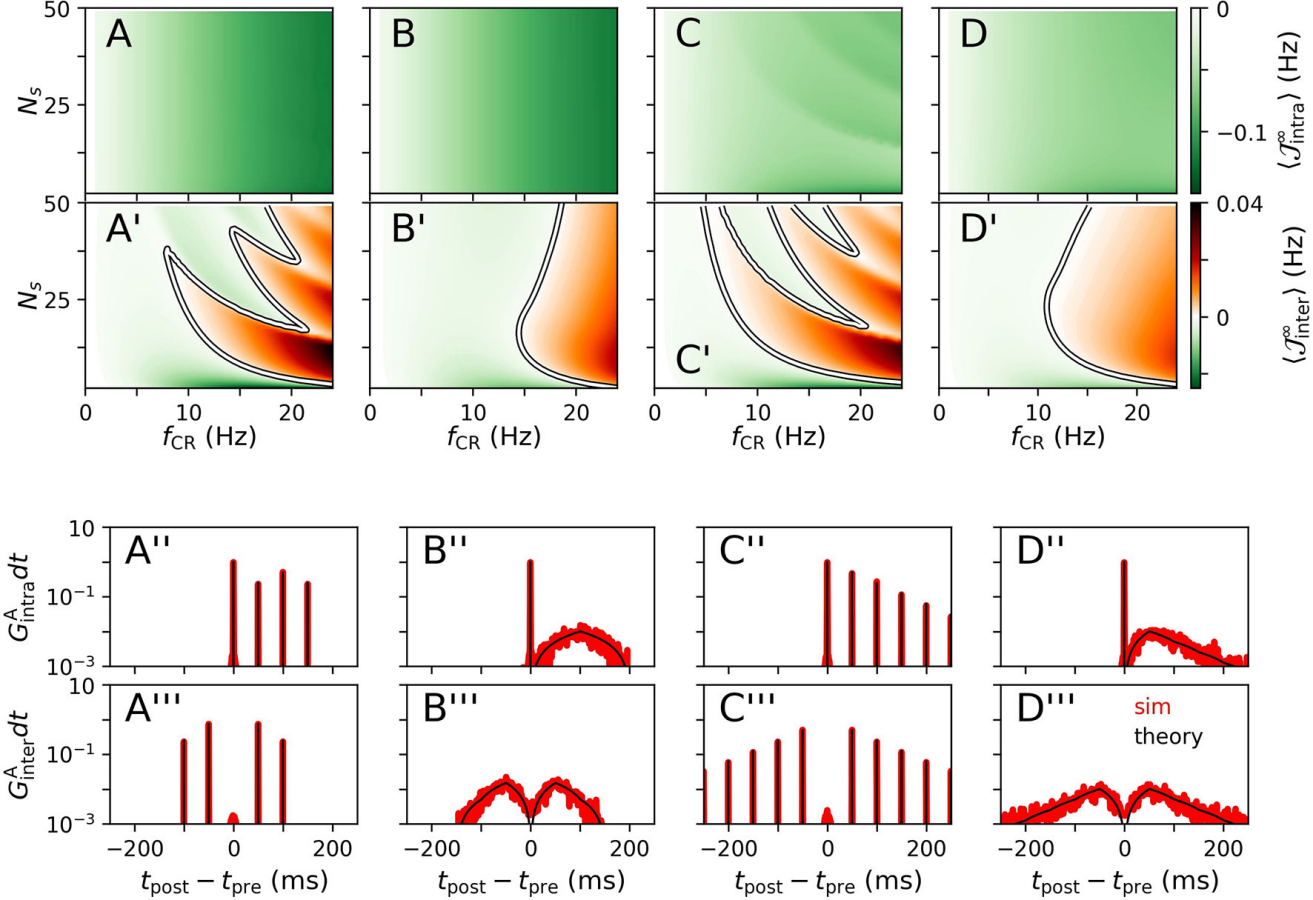
Theoretical estimates of stimulation-induced weight dynamics and distributions of time lags for different CR stimulation patterns. Individual columns correspond to CR **(A)**, NCR **(B)**, SCR **(C)**, and SNCR **(D)** stimulation patterns. Panels show the estimated mean rates of weight change $\langle J_{X}^{\infty }\rangle$, Equation (4), for intrapopulation **(A–D)** and interpopulation synapses **(A'–D')**, respectively. Here, X = “intra,” “inter” marks the considered type of synapses. White curves mark zero contour lines and indicate the boundary between strengthening ($\langle J_{X}^{\infty }\rangle >0$) and weakening of synapses ($\langle J_{X}^{\infty }\rangle <0$). Corresponding estimates for the distributions of time lags that lead to weight updates $G_{X}^{\text{A}}\left(t\right)$ (black), Equation (5), are compared to simulation results (red) in **(A”–D”)** for intrapopulation synapses and in **(A”'–D”')** for interpopulation synaspes, respectively. In **(A”–D”,A”'–D”')**, we set *N*_s_ = 2 and *f*_CR_ = 10 Hz. Networks were simulated for 90 s of ongoing stimulation. Time lags have been recorded from 400 pairs of pre- and postsynaptic neurons. Pairs were sorted according to synapse types “intra” and “inter” and histograms were calculated using a bin size of 1 ms. Theoretical estimates for $J_{X}^{\infty }$ were obtained by numerical calculations of $p_{\text{X}}^{\text{A}}\left(s\right)$, Equation (17). To this end, the time interval [−1, 000, 1, 000] ms was discretized using a binsize of *dt* = 0.01 ms. Then, *G*_X_(*t*) was obtained using Equation (5). To compare theoretical estimates and simulation results, we plotted *G*_X_(*t*)*dt* in **(A”–D”,A”'–D”')** and normalized the histograms such that counts summed up to two. Parameters: *t*_d_ = 3 ms, η = 0.02, τ_+_ = 10 ms, τ_R_ = 4, β = 1.4.

Results for $\langle J_{\text{X}}^{\infty }\rangle$ for CR, NCR, SCR, and SNCR stimulation are shown in Figure 2 for intrapopulation synapses, Figures 2A–D, and interpopulation synapses, Figures 2A'–D', respectively. We find qualitatively different dynamics of intra- and interpopulation synapses. Intrapopulation synapses weaken ($\langle J_{\text{intra}}^{\infty }\rangle <0$) for the considered range of stimulation frequencies *f*_CR_ and numbers of stimulation sites *N*_s_, see Figures 2A–D. In contrast, interpopulation synapses weaken only during slow stimulation with a rather small number of stimulation sites, Figures 2A'–D'. For fast stimulation with larger *N*_s_, interpopulation synapses may strengthen ($\langle J_{\text{inter}}^{\infty }\rangle >0$).

We observe qualitatively different dynamics of interpopulation weights for stimulation patterns with random jitters, i.e., NCRand SNCR, and stimulation patterns with deterministic stimulation times, i.e., CR and SCR, Figures 2A'–D'. For NCR and SNCR stimulation, strengthening of interpopulation synapses ($\langle J_{\text{inter}}^{\infty }\rangle >0$) is observed for fast stimulation, whereas we find a highly nonlinear dependence of the sign of $\langle J_{\text{inter}}^{\infty }\rangle$ for CR and SCR stimulation.

Considering the distributions of time lags that contribute to weight updates $G_{\text{X}}^{\text{A}}\left(t\right)$, Equation (5), we find that stimulation protocols with random jitters, i.e., NCR and SNCR, possess broad distributions spanning a wide range of possible time lags. In contrast, for protocols with deterministic stimulation times, i.e., CR and SCR, $G_{\text{X}}^{\text{A}}\left(t\right)$ is given by a sum of delta-like distributions at integer multiples of *T*_CR_/*N*_s_.

We find that simulated distributions of the time lags between post- and presynaptic spikes, which lead to weight updates, are well-described by theoretical estimates $G_{\text{X}}^{\text{A}}\left(t\right)$ for all four stimulation patterns (for interpopulation synapses, see Figures 2A”–D”, and for intrapopulation synapses see Figures 2A”'–D”'). In particular, smooth distributions of time lags were found for stimulation protocols with temporal randomization, i.e., NCRand SNCR, whereas protocols with deterministic stimulation times led to time lags of multiples of *T*_CR_/*N*_s_.

Based on theoretical predictions for the mean rate of weight change, Figures 2A–D, A'–D', we expect long-lasting desynchronization effects of CR, NCR, SCR, and SNCRin a large portion of the parameter space, spanned by *f*_CR_ and *N*_*s*_.

### 3.2. Acute, Acute After-, and Long-Lasting Effects of Strong Stimulation

**Figure 3:**
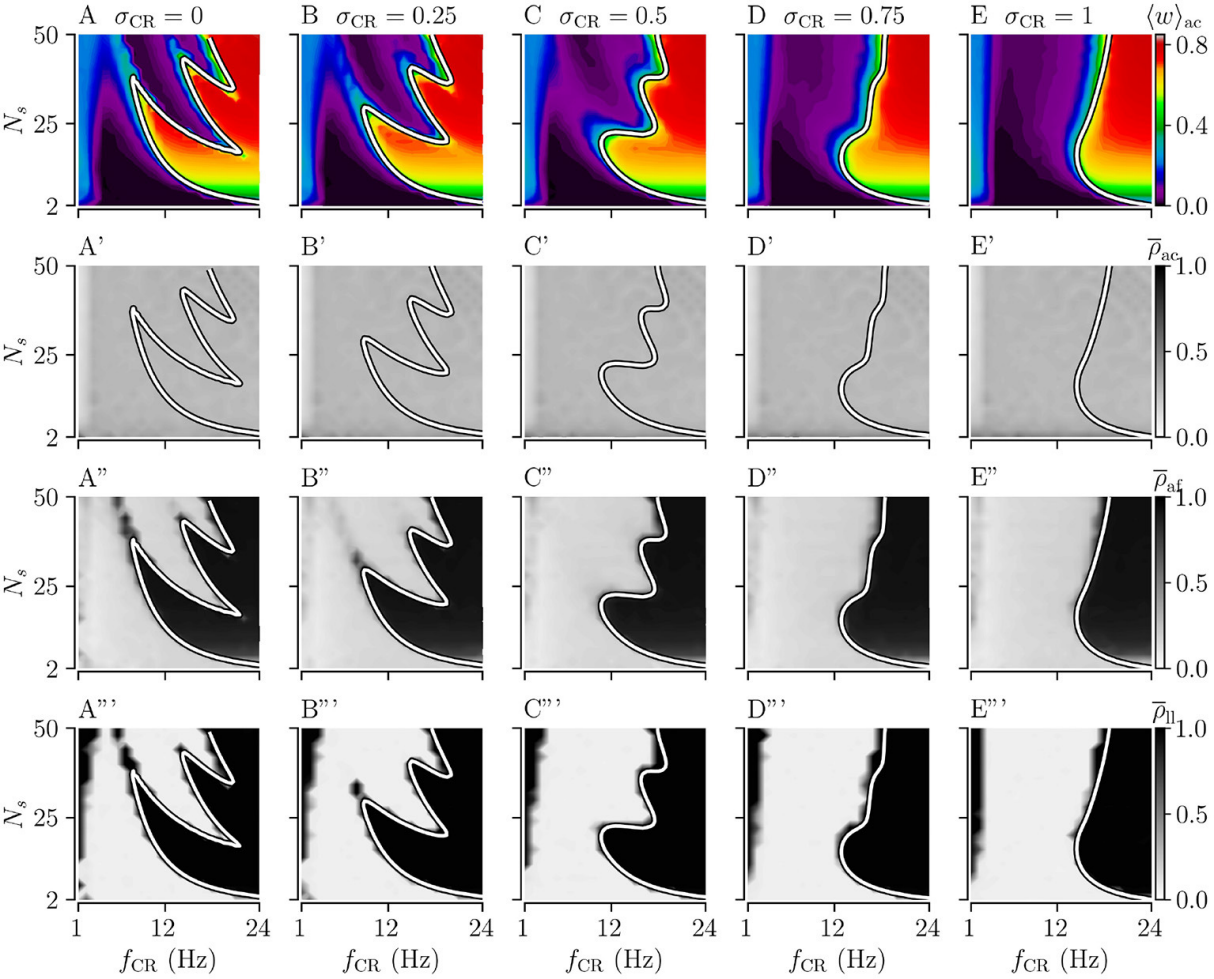
Acute, acute after-, and long-lasting effects of Noisy CR (NCR) with different values of the stimulus jitter, σ_CR_, as a function of the stimulation frequency and the number of stimulation sites for strong stimulation. **(A–E)** Simulation results for the acute mean synaptic weights, 〈*w*〉_ac_, at the end of the 1, 000 s stimulation duration; **(A'–E')** The acute Kuramoto order parameter, ${\bar{\rho }}_{\text{ac}}$, time-averaged over the last 10 s of the stimulation duration; **(A”–E”)** The acute after-effect on synchronization as quantified by the Kuramoto order parameter, ${\bar{\rho }}_{\text{af}}$, time-averaged over a 10 s interval after cessation of the stimulation; **(A”'–E”')** Long-lasting desynchronization effects for respective stimulus jitters, as quantified by the Kuramoto order parameter, Equation (2), averaged over a 10 s interval 1,000 s after cessation of stimulation, ${\bar{\rho }}_{\text{ll}}$. **(A,A',A”,A”')** show results for σ_CR_ = 0 which are similar to Kromer et al. (2020) but for longer stimulation durations. The white curves show theoretical estimates of the boundary between weakening and strengthening of interpopulation synapses, see Figure 2. Parameters: The stimulation duration was set to *T*_stim_ = 1, 000 s and *A*_stim_ = 1.

**Figure 4:**
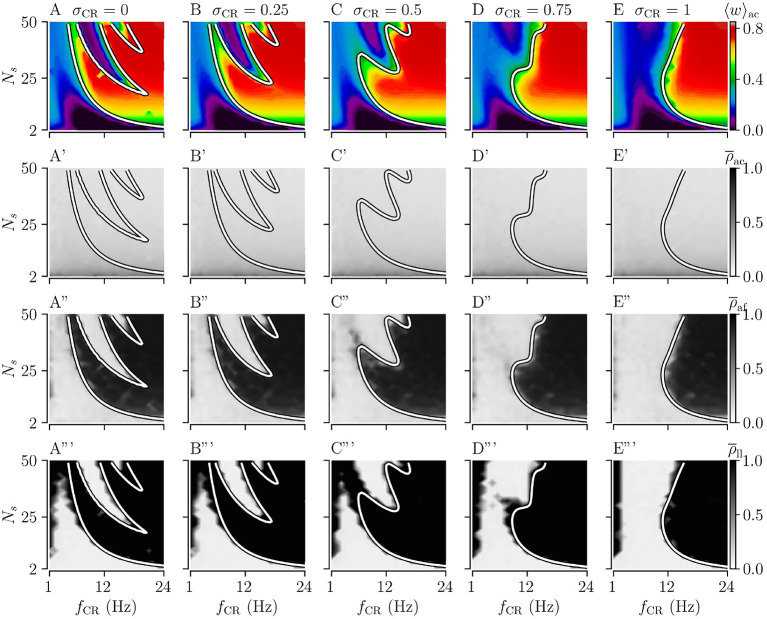
Acute, acute after-, and long-lasting effects of Shuffled Noisy CR (SNCR) for different values of the stimulus jitter, σ_CR_, for strong stimulation as a function of the stimulation frequency and the number of stimulation sites. **(A–E)** Acute mean synaptic weights, 〈*w*〉_ac_; **(A'–E')** The acute Kuramoto order parameter, ${\bar{\rho }}_{\text{ac}}$, time-averaged over the last 10 s of the stimulation; **(A”–E”)** The acute after-effect of stimulation on synchronization as measured by the Kuramoto order parameter, ${\bar{\rho }}_{\text{af}}$, time-averaged over an interval of 10 s right after cessation of the stimulation; **(A”'–E”')** Long-lasting effects of stimulation as quantified by the Kuramoto order parameter, ${\bar{\rho }}_{\text{ll}}$, time-averaged over a 10 s interval 1, 000 s after cessation of stimulation. Parameters: The stimulation duration was set as *T*_stim_ = 1, 000 s and *A*_stim_ = 1.

We study the acute, acute after-, and long-lasting effects of strong stimulation for the four different stimulation patterns. The limit of strong stimulation (*A*_stim_ = 1) resembles the case of stimulation-controlled spiking used during the derivation of theoretical predictions. Simulation results for the acute mean synaptic weight 〈*w*〉_ac_ during NCR stimulation and NSCR stimulation for different values of σ_CR_ are shown in Figures 3A–E, 4A–E, respectively.

First, we consider the extreme cases of deterministic stimulus onset times, σ_CR_=0, and maximum variability of stimulus onset times, σ_CR_ = 1. We find that strong stimulation leads to a reduction of the mean synaptic weight for rather slow stimulation with a small number of stimulation sites. In other parameter regions, the weight dynamics strongly depends on the stimulation pattern. For patterns with deterministic stimulus onset times, i.e., CR (Figure 3A) and SCR (Figure 4A) stimulations, we find a highly nonlinear dependence of the mean synaptic weight on the stimulation frequency and the number of stimulation sites. For CR stimulation, this was previously reported in Kromer et al. (2020). In contrast, for patterns with maximum random jitters, i.e., NCR and SNCR stimulations, we find that stimulation leads to a reduction of the mean synaptic weight for a wide range of stimulation frequencies and numbers of stimulation sites, see Figures 3E, 4E.

In order to study the long-lasting outcome of stimulation, we evaluate the Kuramoto order parameter 1,000 s after cessation of stimulation. Corresponding simulation results are shown in Figures 3A”',E”', 4A”',E”'. We find that stimulation entailed long-lasting desynchronization in regions with considerable weight reduction. This indicates that the system approached the stable desynchronized state after cessation of stimulation. In regions where the mean weight was only slightly reduced, or even increased, during the stimulation, we observe long-lasting synchronization. This indicates that the system reapproached the stable synchronized state for these parameter sets.

To compare theoretical predictions to simulation results, we show the estimated boundary between stimulation-induced weakening and strengthening of interpopulation synapses ($\langle J_{\text{X}}^{\infty }\rangle =0$) in Figures 3, 4. We find that the boundary accurately reproduces the boundary between long-lasting desynchronization and long-lasting synchronization. Deviations occur mainly for low and intermediate stimulation frequencies.

Next, we analyze the degree of stimulation-induced synchronization during stimulation (acute effects), right after cessation of stimulation (acute after-effects), and long after cessation of stimulation (long-lasting effects). Figures 3, 4 show corresponding acute effects (second row), acute after-effects (third row), and long-lasting after effects (fourth row) as quantified by the Kuramoto order parameter, Equation (2), averaged over respective time intervals.

We find that acute stimulation-induced synchrony during NCR stimulation is independent of the stimulation parameters, such as the stimulation frequency, the number of stimulation sites, and the jitter σ_CR_. Throughout the parameter space, NCR stimulation induces acute partial synchronization with ρ_ac_ ≈ 0.3. Only for very slow stimulation (*f*_CR_ ≈ 1 − 2 Hz), NCR stimulation induces acute desynchronization. Remarkably, as soon as stimulation ceases, the degree of synchronization changes rapidly and becomes determined by the underlying network connectivity, i.e., strong synaptic connections lead to synchronization and weak synaptic connections to desynchronization, see the third row of Figure 3.

Similarly, for SNCR stimulation, we find acute partial synchronization. However, the degree of in-phase synchronization attains lower values for larger *N*_s_, see Figure 4. As for NCR stimulation, the degree of synchronization changes rapidly after cessation of SNCR stimulation and attains the values determined by the underlying network connectivity. Of note, for all stimulation patterns shown in Figures 3, 4, for very low stimulation frequencies, *f*_CR_ → 1 Hz, there are no long-lasting effects for most values of *N*_s_. There, the CR stimulation frequency, *f*_CR_, is much smaller than the frequency of the synchronized rhythm. Hence even if the stimulation potentially can decouple the network, a much longer stimulation duration is needed.

### 3.3. Effect of Jitter on Long-Lasting Effects

Next, we consider the effect of σ_CR_, quantifying the variability of stimulus onset times. We perform simulations for intermediate values of 0 < σ_CR_ < 1 and evaluate the theoretical prediction of the boundary between strengthening and weakening of interpopulation weights ($\langle J_{\text{X}}^{\infty }\rangle =0$) using Equations (4) and (5), and the results for the respective stimulation patterns given in section 2. Results for NCR stimulation and SNCR are given in Figures 3, 4, respectively.

We find that for both NCR and SNCR stimulations, the nonlinear dependence of the mean synaptic weight on the stimulation frequency and the number of stimulation sites becomes less pronounced as σ_CR_ increases, see Figures 3, 4. This trend is well reproduced by our theory. We observe a similar trend for the degree of long-lasting desynchronization as quantified by the Kuramoto order parameter, see the fourth row of Figures 3, 4.

**Figure 5:**
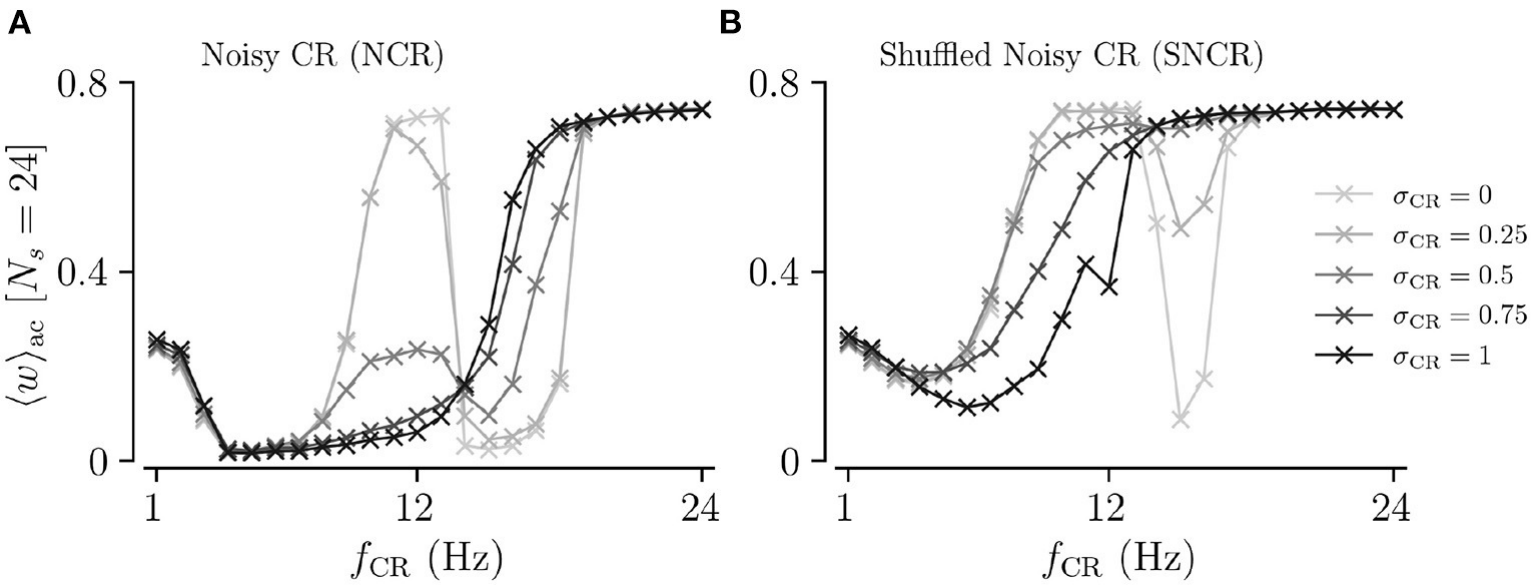
Acute mean synaptic weight for five values of the stimulus jitter, σ_CR_, as a function of the stimulation frequency for *N*_s_ = 24. **(A,B)** show results for Noisy CR (NCR) and Shuffled Noisy CR (SNCR), respectively. These graphs represent horizontal lines at *N*_s_ = 24 in Figures 3, 4, respectively. Parameters are the same as in Figures 3, 4.

To directly compare the mean synaptic weights for different values of σ_CR_, we fix the number of stimulation sites to *N*_s_ = 24. Results for the mean synaptic weight for different σ_CR_ as a function of the stimulation frequency are plotted in Figure 5. We find that the effect of variability of stimulus onset times on the acute mean synaptic weight depends on the stimulation frequency. In particular, we find that increasing σ_CR_ has only a minor effect on the acute mean weight for NCR stimulation with low stimulation frequencies, see Figure 5A. In contrast, it leads to a significant reduction of the acute mean weight for intermediate stimulation frequencies, (*f*_CR_ ≈ 12 Hz), whereas it leads to an increase of the acute mean weight for higher stimulation frequencies, see Figure 5A. For SNCR stimulation, we find qualitatively similar results, however, these trends occur at slightly lower stimulation frequencies, see Figure 5B.

Of particular interest for clinical application is to provide parameter ranges for which adding random jitters to the stimulus onset times might improve the performance of the stimulation. In order to derive such parameter ranges, we consider the difference of the acute mean synaptic weight for deterministic stimulus onset times 〈*w*〉_ac_(CR), and the acute mean synaptic weight for maximum variability (σ_CR_ = 1) 〈*w*〉_ac_(NCR). We also calculate the corresponding difference for SCR and SNCR.

**Figure 6:**
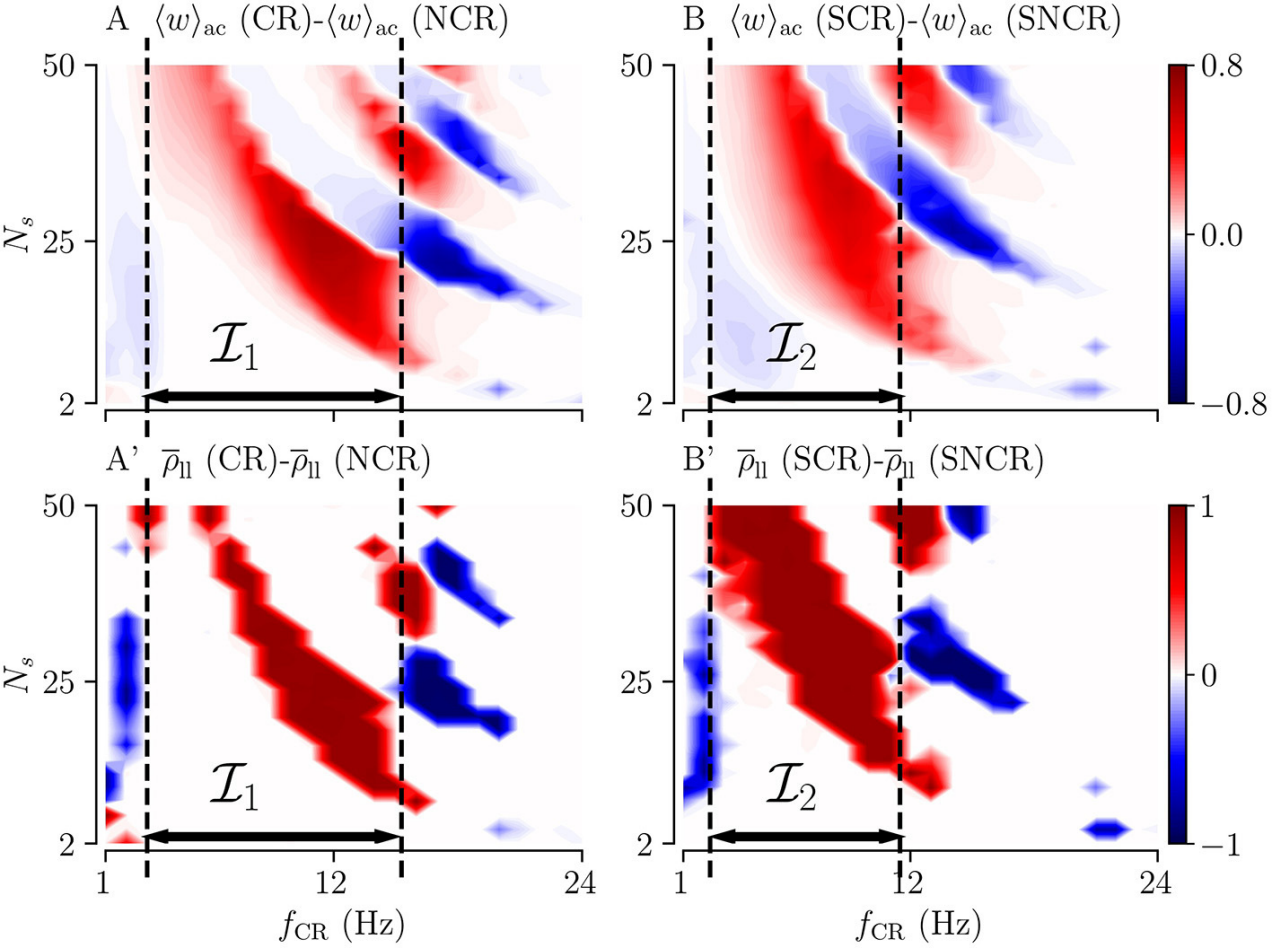
Differences between the outcomes of stimulation with deterministic stimulus onset times (CR/SCR) and NCR/SNCR stimulation with maximum jitter, σ_CR_ = 1. The difference maps for the acute mean weight **(A,B)** and for the long-lasting Kuramoto order parameter **(A',B')**. Parameter regions where NCR/SNCR led to smaller mean weight/values of the long-lasting Kuramoto order parameter compared to CR/SCR are marked red. Dashed vertical lines enclose the largest continuous range of stimulation frequencies where NCR/SNCR stimulation with maximum jitter led to similar or better outcome than CR/SCR stimulation. These frequency ranges are referred to as $I_{1}$, and $I_{2}$ in the text. Data are taken from panels A,E (acute mean weight) and A”',E”' (long-lasting Kuramoto order parameters) of Figures 3, 4, respectively.

Results are shown in Figures 6A,B for NCR and SNCR stimulation, respectively. In the red regions in Figure 6, jitter improves the reduction of the mean synaptic weight during stimulation. Accordingly, we calculate the differences between the Kuramoto order parameters, ${\bar{\rho }}_{\text{ll}}$, quantifying the effect of jitter on the degree of long-lasting desynchronization. Corresponding results are shown in Figures 6A',B'.

The simulation results in Figure 6 show that adding random jitters to the stimulus onset times during CR and SCR improves the performance of these stimulation patterns for intermediate frequencies. Corresponding frequency intervals $I_{\text{1}}$ (NCR) and $I_{\text{2}}$ (SNCR) are shown in Figure 6. However, for very low and high frequencies, adding random jitters tends to worsen the outcome of the stimulation.

### 3.4. Acute and Long-Lasting Effects of Weak Stimulation

Next, we consider weak stimulation using the four different stimulation patterns; CR, SCR, and their noisy counterparts with maximum jitter, σ_CR_ = 1. To this end, we set *A*_stim_ = 0.1 and perform a similar analysis as in the previous section.

**Figure 7:**
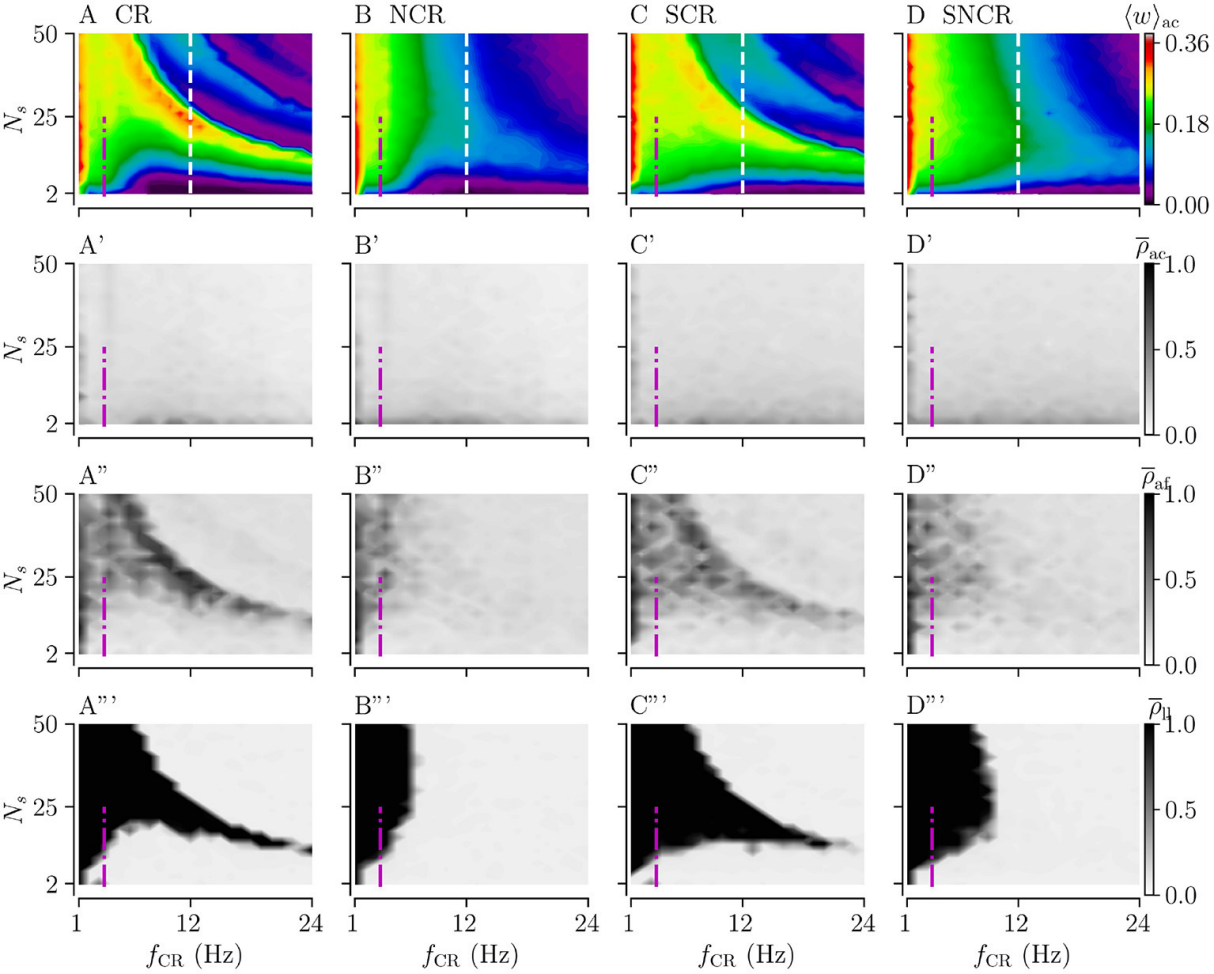
Acute, acute after-, and long-lasting effects of weak stimulation for the four multisite stimulation protocols. Upper **(A–D)** show simulation results for the acute mean weight, 〈*w*〉_ac_; **(A'–D')** show the acute Kuramoto order parameter, ${\bar{\rho }}_{\text{ac}}$, time-averaged over the last 10 s of the stimulation duration; **(A”–D”)** show the acute after-effect measured by the Kuramoto order parameter, ${\bar{\rho }}_{\text{af}}$, 10 s after cessation of the stimulation; and the bottom panels **(A”'–D”')** show the results for the Kuramoto order parameter, ${\bar{\rho }}_{\text{ll}}$, time-averaged over a 10 s time interval 1, 000 s after cessation of stimulation. Low values of the Kuramoto order parameter indicate desynchronized activity while high values refer to pronounced in-phase synchronization. The white vertical lines in **(A–D)** mark a stimulation frequency of *f*_CR_ = 12 Hz for which we present a detailed analysis of the influence of the stimulation duration *T*_stim_ in Figure 8. The frequency of the original synchronous rhythm is approximately 3.5 Hz and it is shown by the magenta dot-dashed vertical lines. Acute mean weights are measured at the end of *T*_stim_ = 500 s stimulation period. Parameters: *A*_stim_ = 0.1.

Figures 7A–D show simulation results for the acute mean weight 〈*w*〉_ac_ during weak stimulation for the four different stimulation patterns. We find that stimulation leads to a reduction of the mean synaptic weight (prior to stimulation 〈 *w* 〉 ≈ 0.38) in the major part of the parameter space spanned by the stimulation frequency and the number of stimulation sites. In contrast to strong stimulation, weak stimulation also leads to weight reduction for fast stimulation with large numbers of stimulation sites. In the maps of Figures 7A–D, the red colors indicate that the value of the mean synaptic weight is close to that prior to stimulation in the stable synchronized state. Overall weight reduction during weak stimulation is more pronounced for sufficiently fast stimulation.

Considering the differences between the four stimulation protocols, we find that protocols with deterministic stimulation times, i.e., CR and SCR stimulations, do not lead to a substantial reduction of the mean synaptic weight for the intermediate number of stimulation sites and a wide range of stimulation frequencies. In contrast, protocols with random jitters, i.e., NCR and SNCR stimulations, lead to a reduction of the mean synaptic weight for sufficiently fast stimulation, *f*_CR_ ≿ 6 Hz for NCR and *f*_CR_ ≿ 10 Hz SNCR stimulation, across the range of considered numbers of stimulation sites, see Figures 7A–D.

For a vast range of stimulation frequencies, we find a nonlinear dependence of 〈*w*〉_ac_ on the number of stimulation sites. While 〈*w*〉_ac_(*N*_s_) expresses multiple extrema for constant *f*_CR_ for CR and SCR stimulations, we observe only one maximum for NCR and SNCR stimulations, see Figures 7A–D.

Next, we consider the acute, acute after-, and long-lasting effects of stimulation on synchronization. Simulation results are shown in Figure 7. Similar to the case of strong stimulation, we find acute partial synchronization during weak stimulation, see the second row of Figure 7. As stimulation ceases, the network approaches the state determined by the network connectivity, i.e., synchronized activity for strong and desynchronized activity for weak connections, see the third row of Figure 7. Remarkably, in parts of the parameter space where synaptic weights have not been reduced completely during the stimulation, we find that the Kuramoto order parameter increases right after cessation of stimulation, compare rows two and three of Figure 7. As one can see from the acute after-effects, (see Figures 7A”–D”), we find pronounced in-phase synchronization in parameter regions where stimulation did not lead to a substantial reduction of the mean synaptic weight, compare Figures 7A–D, A”'–D”'. As a consequence, stimulation protocols with random jitters, i.e., NCR and SNCR stimulation, lead to long-lasting desynchronization, ${\bar{\rho }}_{\text{ll}}\approx 0$, in a bigger portion of the parameter space than stimulation protocols without random jitters, i.e., CR and SCR stimulations.

### 3.5. Required Stimulation Duration for Long-Lasting Desynchronization

We study the influence of the stimulation duration *T*_stim_ on the acute and long-lasting outcome of weak stimulation with the four different stimulation patterns. To this end, we fix the stimulation frequency to *f*_CR_ = 12 Hz, see dashed vertical lines in Figures 7A–D. We deliver stimulation for a stimulation duration *T*_stim_ and record the acute mean synaptic weight 〈*w*〉_ac_. Then, we turn off the stimulation and continue the simulation for 1, 000 s to evaluate long-lasting effects using ${\bar{\rho }}_{\text{ll}}$.

**Figure 8:**
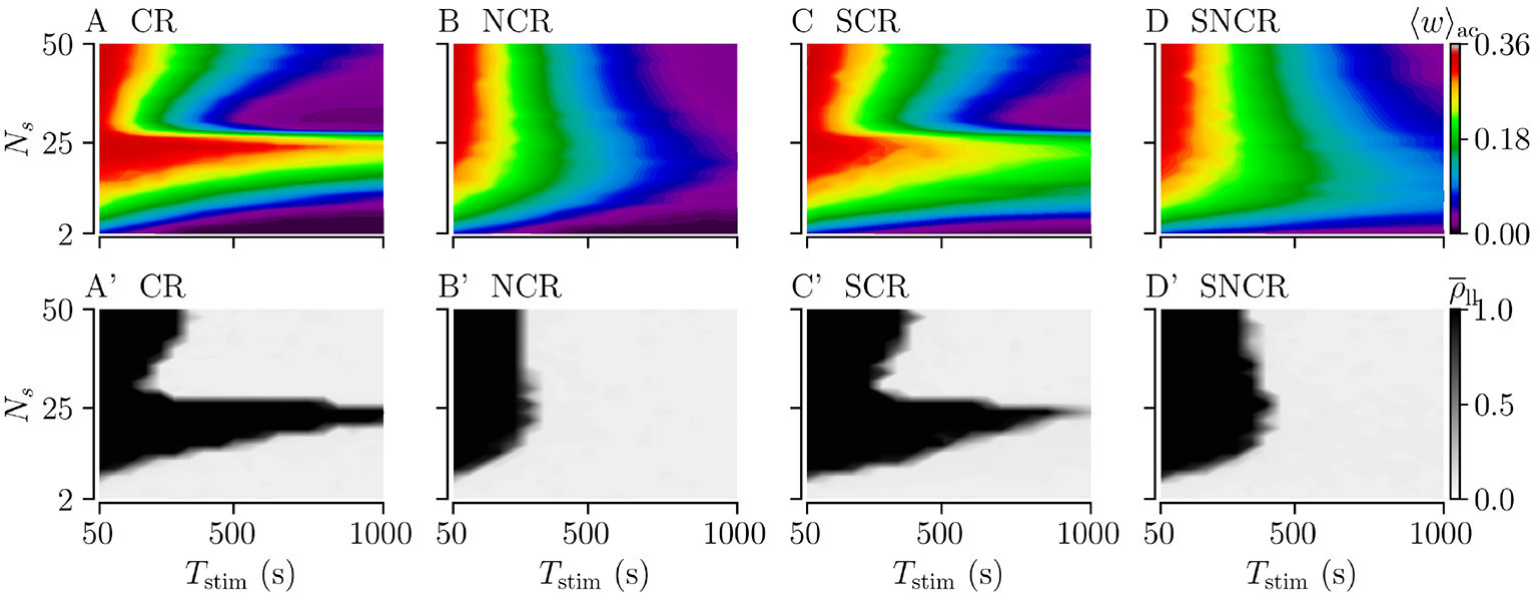
Acute and long-lasting effects of stimulation for different stimulation durations. **(A–D)** acute mean synaptic weight, 〈*w*〉_ac_, at the end of a stimulation period *T*_stim_ for different numbers of stimulation sites *N*_s_. **(A'–D')** Long-lasting desynchronization effects quantified by the Kuramoto order parameter, ${\bar{\rho }}_{\text{ll}}$, Equation (2), recorded 1, 000 s after a stimulation period of duration *T*_stim_. Low values indicate desynchronized spiking activity and high values in-phase synchronization of neuronal spiking. Parameters: *A*_stim_ = 0.1 and *f*_CR_ = 12 Hz.

Figures 8A–D shows simulation results for the acute mean weight 〈*w*〉_ac_ recorded after the stimulation duration *T*_stim_. We find that the mean weight 〈*w*〉_ac_ reduces rapidly for small numbers of stimulation sites, *N*_s_ < 10. For larger *N*_s_, the evolution of 〈*w*〉_ac_(*T*_stim_) depends on the stimulation protocol. For CR and SCR stimulation, 〈*w*〉_ac_(*T*_stim_) reduces rather rapidly for a narrow range of large 26 < *N*_s_ < 40, whereas it reduces slowly for others *N*_*s*_. In contrast, the reduction of 〈*w*〉_ac_(*T*_stim_) only slightly depends on *N*_s_ for NCR and SNCR stimulation.

### 3.6. Long-Lasting Desynchronization Effects Depend on Stimulation Strength

Typically, in clinical trials, the stimulation frequency and strength can be adjusted more readily, whereas the number of stimulation sites is constrained by anatomical and physiological features of the target area. Accordingly, we study the effect of the stimulation strength, *A*_stim_, and the stimulation frequency, *f*_CR_, for fixed numbers of stimulation sites. We study the long-lasting desynchronization effects, as quantified by the Kuramoto order parameter $\bar{\rho_{\text{ll}}}$, for CR and SCR and their noisy counterparts with maximum jitter, σ_CR_ = 1. For *N*_s_ = 4, 8, 16, and 24, we vary the stimulation frequency and strength.

**Figure 9:**
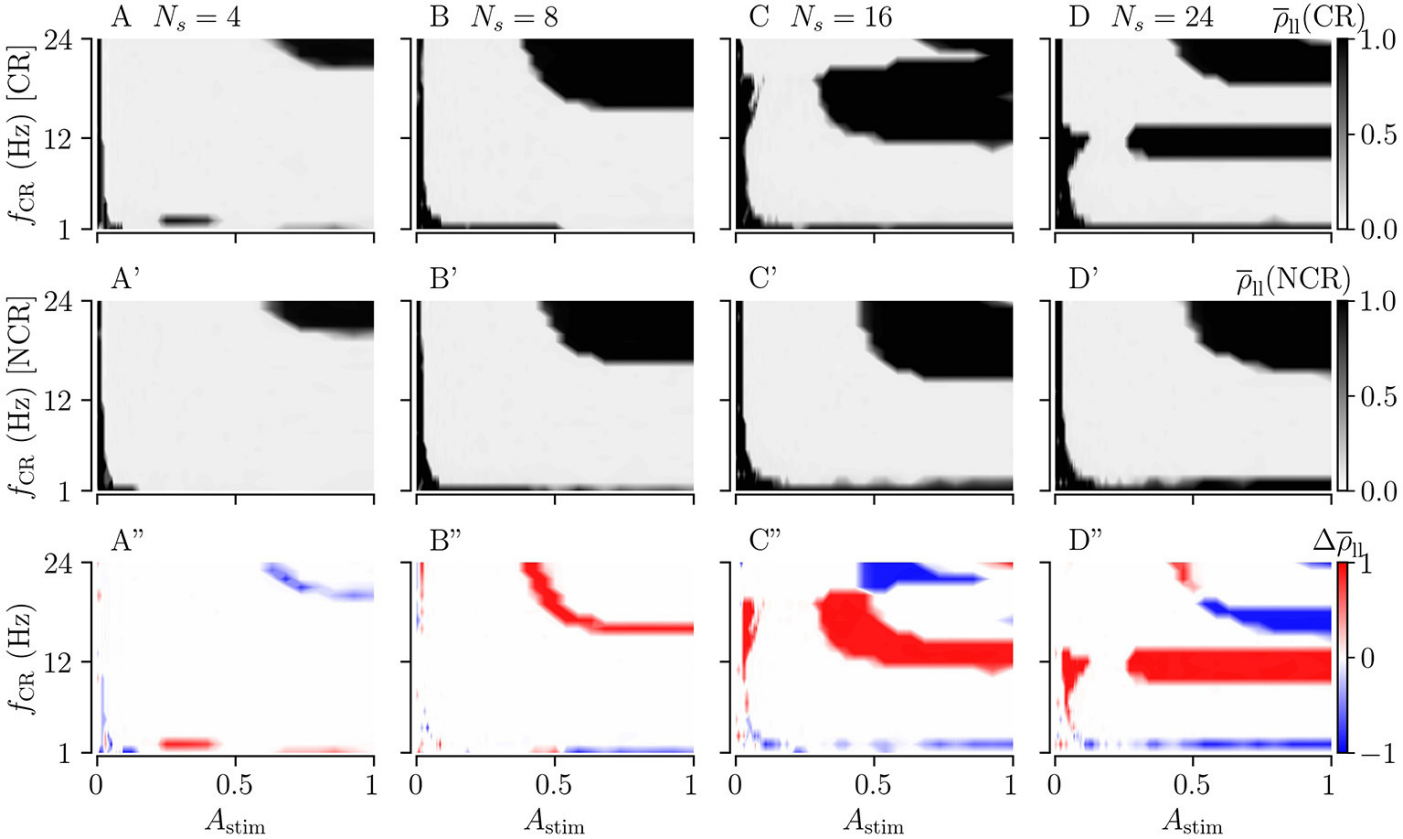
Long-lasting effects of CR and NCR stimulations as a function of CR frequency, *f*_CR_, and simulation strength, *A*_stim_. **(A–D)** The long-lasting Kuramoto order parameter for CR stimulation as a function of stimulation frequency and stimulation strength for *N*_s_ = 4, 8, 16, and 24. **(A'–D')** Same as the top row but for NCR with σ_CR_ = 1. **(A”–D”)** The difference between the long-lasting Kuramoto order parameter for CR and NCR, $\Delta {\bar{\rho }}_{\text{ll}}={\bar{rho}}_{\text{ll}}\left(\text{CR}\right)-{\bar{rho}}_{\text{ll}}\left(\text{NCR}\right)$. In the red colored parameter regions, the Kuramoto order parameter for CR was larger than that for NCR, in the blue colored regions CR led to long-lasting desynchronization whereas NCR did not. The columns correspond to different numbers of stimulation sites, *N*_s_ = 4, 8, 16, and 24, respectively.

Figures 9A–D shows the long-lasting desynchronization effects for CR, and Figures 9A'–D' shows the same for NCR. The third row in Figures 9A”–D” shows the difference between the long-lasting Kuramoto order parameters of CR and NCR.

For weak stimulation strengths (*A*_stim_ → 0) the stimulation is ineffective in inducing long-lasting desynchronization. For moderate stimulation strengths, both CR and NCR lead to desynchronization over a wide range of stimulation frequencies. However, for NCR, the range of effective moderate simulation strengths is larger compared to CR, as can be seen in the second row of Figure 9. By increasing the stimulation strength (*A*_stim_ → 1), we approach the limit of strong stimulation (predicted by our theory, see Figure 2). Here, we find several frequency intervals in which stimulation does not lead to long-lasting desynchronization (see black regions in Figure 9). Remarkably, we find only one of these frequency intervals for NCR, whereas several intervals occur for CR. Put differently, random jitters remove the nonlinearities in the parameter space (Compare A–D with A'–D' in Figure 9). We find that the parameter region in which stimulation leads to long-lasting desynchronization is bigger for smaller numbers of stimulation sites, i.e., *N*_s_ = 4.

**Figure 10:**
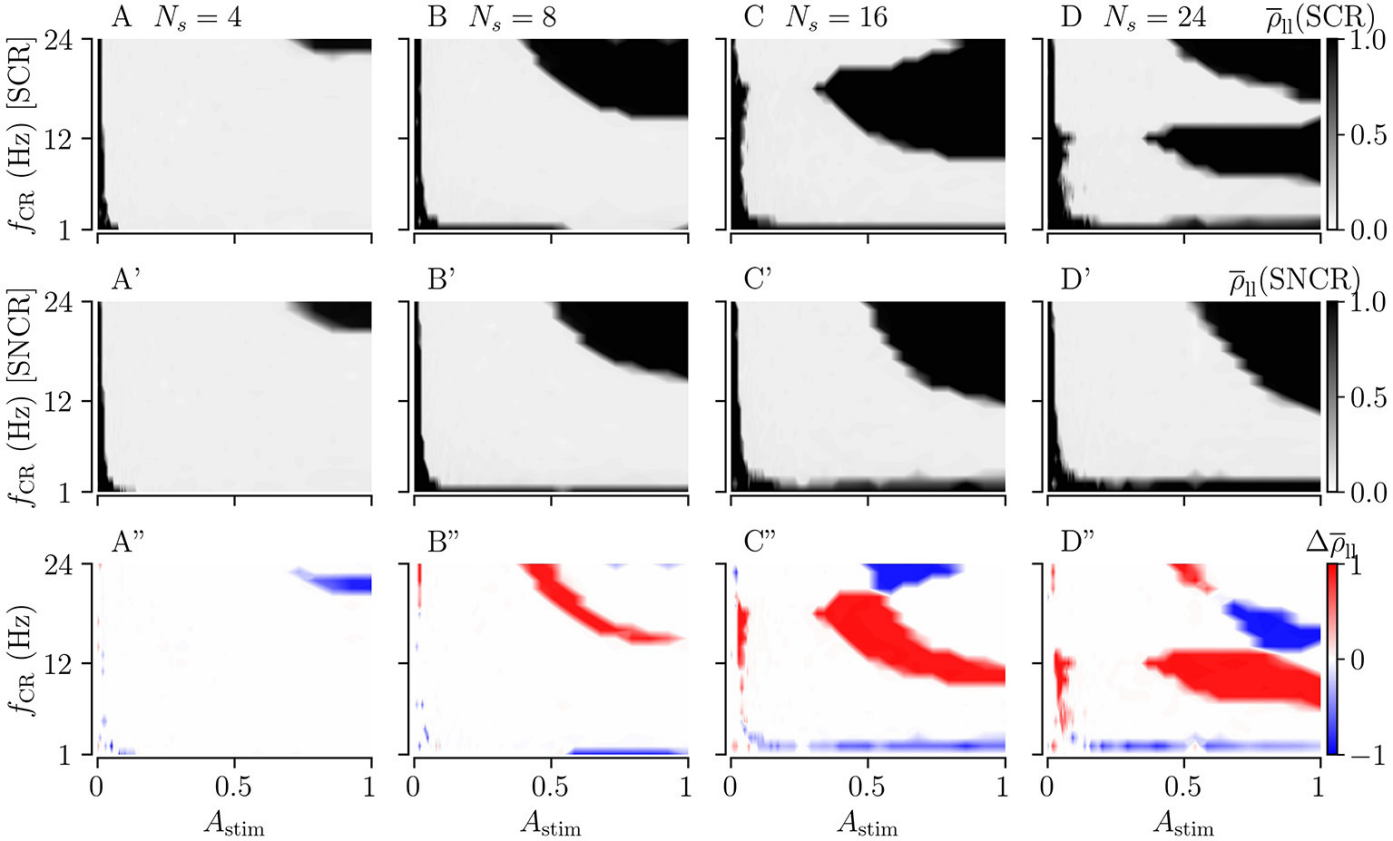
Long-lasting effects of SCR and SNCR stimulation as a function of CR frequency, *f*_CR_, and simulation strength, *A*_stim_. Panels show the long-lasting Kuramoto order parameter for SCR **(A–D)** and for SNCR **(A'–D')**. **(A”–D”)** show the difference between the long-lasting Kuramoto order parameter for SCR and SNCR, $\Delta {\bar{\rho }}_{\text{ll}}={\bar{rho}}_{\text{ll}}\left(\text{SCR}\right)-{\bar{rho}}_{\text{ll}}\left(\text{SNCR}\right)$. This figure is similar to Figure 9 but for SCR and SNCR.

Figure 10 shows the long-lasting effect for SCR and SNCR. Here, we find qualitatively similar results as in Figure 9. Comparing the results for CR and SCR, we find that SCR performs slightly better than CR at low stimulation frequencies (*f*_CR_ = 1 − 3 Hz) (see Figures 9A, 10A). In the limit of strong stimulation, frequency intervals in which stimulation does not lead to long-lasting desynchronization effects are larger for SCR and SNCR than for CR and NCR. However, this reverses if the stimulation strength is reduced. Thus, shuffling increases the range of effective stimulation frequencies for moderate to strong stimulation amplitudes.

## 4. Discussion

In the present paper, we analyze the acute, acute after-, and long-lasting effects of randomized coordinated reset (CR) stimulation patterns on plastic neuronal networks. CR stimulation has been used in preclinical *in vitro* studies (Tass et al., 2009), preclinical *in vivo* studies (Tass et al., 2012b; Wang et al., 2016; Ho et al., 2021), as well as in clinical studies (Adamchic et al., 2014; Syrkin-Nikolau et al., 2018; Pfeifer et al., 2021) to induce acute and long-lasting desynchronization effects as well as symptom relief in the context of epilepsy, Parkinson's disease, and binge alcohol drinking. We computationally study the consequences of a reduction of temporal correlations between stimulus delivery times and a reduction of spatial correlations in the stimulus pattern on the efficacy of stimulation. A reduction of temporal correlations is achieved by adding random jitters to the deterministic stimulus delivery times of the original CR pattern. We denote the resulting stimulation pattern as CR with random jitters (NCR). A corresponding stimulation pattern has been used in a recent clinical study on vibrotactile stimulation of Parkinson's patients; where a long-lasting cumulative reduction of motor symptoms was observed (Pfeifer et al., 2021). The reduction of spatial correlations is achieved by shuffling the sequence of stimulated subpopulations of the original CR pattern. The corresponding stimulation pattern is referred to as shuffled CR (SCR). Lastly, we refer to a CR pattern with both random jitters and shuffling as shuffled noisy CR (SNCR). Corresponding stimulation patterns are illustrated in Figure 1. Our detailed theoretical and computational analysis reveals a significant increase in parameter robustness of long-lasting effects due to random jitters for intermediate stimulation frequencies, whereas shuffling reduces parameter robustness.

First, we consider the limit of strong stimulation for which we accurately predict the distribution of time lags between post- and presynaptic neurons by extending a theoretical framework previously presented in Kromer and Tass (2020), see Figure 2. In particular, we find marked differences between the stimulation-induced dynamics of intrapopulation and interpopulation synapses. Here, intrapopulation synapses refer to synapses that connect neurons at the same stimulation site, while interpopulation synapses connect neurons at different stimulation sites.

For all considered stimulation patterns, we found a stimulation-induced weakening of intrapopulation synapses. This is in accordance with previous results on the stimulation-induced weight dynamics in plastic neuronal networks (Kromer and Tass, 2020; Kromer et al., 2020; Khaledi-Nasab et al., 2021a,b; Pfeifer et al., 2021). These studies found that the dynamics of intrapopulation synapses is dominated by an effect called *decoupling through synchrony* (Lubenov and Siapas, 2008; Knoblauch et al., 2012). For sharp distributions of spike response times, this effect leads to a reduction of synaptic weights between simultaneously stimulated neurons in networks with axonal delays. For sufficiently long axonal delays, stimulus-elicited presynaptic spikes arrive after the postsynaptic ones which leads to pronounced synaptic depression. In our setup, this effect strongly contributes to the dynamics of the mean synaptic weight for small numbers of stimulation sites *N*_s_, i.e., when the portion of intrapopulation synapses is high. Consequently, we observe a rapid reduction of the mean synaptic weight for small *N*_s_, see, for instance, Figure 8.

In contrast, the dynamics of interpopulation synapses is more complex and it depends on the stimulation patterns. A detailed analysis for regular CR has been provided in Kromer et al. (2020). The authors revealed a nonlinear dependence of the mean rate of weight change on the stimulation frequency, *f*_CR_, and the number of stimulation sites used for stimulus deliveries, *N*_s_. These nonlinearities result from a delay-induced effect that leads to a change in the order of postsynaptic spikes and presynaptic spike arrival times whenever *k*/*f*_CR_*N*_s_ < *t*_*d*_, i.e., when spikes triggered by the next stimulus tend to occur before stimulus-triggered presynaptic spikes arrive. Here *k* is a natural number referring to how many stimuli may be delivered during a single delay time *t*_*d*_. This effect results in the complex pattern of synaptic weakening ($\langle J_{\text{inter}}^{\infty }\rangle <0$) and synaptic strengthening ($\langle J_{\text{inter}}^{\infty }\rangle >0$) of interpopulation synapses, see Figure 2A'. We find the corresponding pattern for SCR stimulation, i.e., for CR with shuffled sequence of stimulation sites, see Figure 2C'. However, these nonlinearities disappear if random jitters are added, i.e., for NCR or SNCR stimulation. For these stimulation patterns, we observe one continuous region in the parameter space where interpopulation weights reduce. This happens because the condition *k*/*N*_s_*f*_CR_ < *t*_*d*_ is only satisfied by small portions of stimuli, due to random jitters. Considering these portions in our theory led to an accurate prediction of regions with synaptic strengthening and weakening for these patterns, see Figures 2B',C'.

Using our theoretical framework, we were able to accurately predict the distribution of time lags between post- and presynaptic spikes for strong stimulation. Results for intrapopulation synapses are presented in Figures 2A”–D” and results for interpopulation synapses in Figures 2A”'–D”'. Stimulus patterns with deterministic stimulus delivery times, i.e., CR and SCR, cause distributions with several peaks at integer multiples of 1/*N*_s_*f*_CR_, i.e., multiples of the minimal inter-stimulus interval, see Figures 2A”,A”',C”,C”'. Similar distributions have been reported in Kromer and Tass (2020); Kromer et al. (2020) for CR stimulation. Kromer et al. (2020) pointed out that stimulation patterns causing such distributions of time lags require an adjustment of the inter-stimulus intervals to the STDP time scales τ_+_ and τ_−_. Random jitters, however, lead to a smoothening of these distributions, see results for NCR and SNCR in Figure 2 for the maximum jitter, σ_CR_ = 1. In Kromer and Tass (2020), a smoothed distribution of time lags was also obtained for random reset (RR) stimulation, which combines temporal and spatial randomization by delivering stimuli at random times to randomly selected subpopulations. The authors compared the performance of RR stimulation for different STDP functions, including the one used in the present paper, Equation (1), and suggested that stimulation patterns that cause smoothed distributions of time lags, may lead to weight reduction that is more robust with respect to changes of the stimulation frequency. Our results support this suggestion. In particular, we found that NCR and SNCR stimulations lead to a pronounced weakening of interpopulation synapses in a large portion of the parameter space spanned by the stimulation frequency and the number of stimulation sites used for stimulus deliveries, see Figures 2–4.

We found that stimulation that leads to a pronounced weakening of interpopulation synapses may entail long-lasting desynchronization, see Figures 3, 4. In particular, for different values of the jitter, our theory accurately predicts the boundary between regions of stimulation parameters that lead to long-lasting desynchronization and those that lead to long-lasting synchronization. We find that deviations mainly occur for slow stimulation, i.e., where stimuli are administered at a slower pace than the original synchronous rhythm (≈3.5 Hz) and right next to the boundary, where weight reduction occurs at low rates and longer stimulation durations are required to drive the network into the attractor of the stable desynchronized state. In Figures 3, 4, we also found deviations for high frequencies (*f*_CR_ > 8 Hz) and large numbers of stimulation sites. These are expected as our theory only considers first-order corrections, i.e., it is restricted to the case that only postsynaptic spikes (triggered by the next stimulus) may arrive before presynaptic spikes (triggered by the current stimulus). For fast stimulation that uses a large number of stimulation sites, however, multiple stimuli may trigger postsynaptic spikes before the presynaptic spike arrives at the postsynaptic neuron.

In contrast to the long-lasting effects of stimulation, we didn't find a strong parameter dependence of the degree of acute synchronization (see the second row of Figures 3, 4). During strong stimulation, the degree of synchronization is determined by the stimulus pattern rather than the synaptic weights. In particular, in the limit of strong stimulation, the degree of acute synchronization is independent of the stimulation frequency as long as the latter is fast compared to the synchronous rhythm, see the second row of Figures 3, 4. For NCR stimulation with various jitters, we find acute partial synchronization which is in accordance with previous results on acute partial synchronization during CR stimulation, see Figure 4 in Kromer et al. (2020). For slower NCR stimulation, we find a reduction of the acute Kuramoto order parameter indicating acute desynchronization. This is due to slow but strong stimulus deliveries that perturb the original synchronous rhythm. During SNCR stimulation, we also observed partial acute synchronization, however, the Kuramoto order parameter depends on the number of stimulation sites, see the second row of Figure 4. A similar effect was observed in studies on strong RR stimulation (Khaledi-Nasab et al., 2021b). There it was argued that a low number of stimulation sites leads to synchronous spiking responses of macroscopic neuronal subpopulations. Strong RR and SNCR stimulation possess qualitatively similar correlations between stimulus delivery times compare Equations (9, 10) in Khaledi-Nasab et al. (2021b) with Equations (12, 13) in the present paper. Therefore, we expect a similar dependence on the number of stimulation sites for strong stimulation. For weak stimulation, we find a slight dependence on the stimulation frequency. In particular, partial synchronization during CR and NCR stimulation attains a maximum when *f*_CR_ is close to the frequency of the synchronous rhythm (The synchronous rhythm is shown by magenta vertical lines in Figure 7). This is similar to previous computation results on CR stimulation, where weak CR stimulation was inefficient when the stimulation frequency attained multiples of the frequency of the underlying synchronous rhythm (Kromer and Tass, 2020).

In contrast to the acute synchronization effects, the acute after-effects shown in the third rows of Figures 3, 4, 7, showed a great correspondence to the long-lasting effects. Note that in the case of weak stimulation, a slight increase of the Kuramoto order parameter was observed in regions where the synaptic weights were only slightly reduced during stimulation. Thus, based on the results in our computational model, we find that acute after-effects might be more suitable for predicting long-lasting effects than acute effects. This aligns with previous studies on acoustic CR for the treatment of tinnitus; where stimulation-induced acute and acute after-effects (Adamchic et al., 2017), as well as long-lasting effects (Tass et al., 2012a) were studied. The authors suggested that significant acute after-effects might be predictive of long-lasting symptom relief (Adamchic et al., 2017).

Remarkably, we find that both the synaptic weight reduction during and the long-lasting desynchronization by weak stimulation are more robust with respect to changes of the stimulation frequency and the number of stimulation sites than those of strong stimulation. In particular, for weak stimulation, we found that all four stimulation patterns cause a pronounced synaptic weight reduction and long-lasting desynchronization for high stimulation frequencies and large numbers of stimulation sites. This is in line with the results of Kromer et al. (2020), who observed this for the CR stimulation pattern. They argued that weak stimuli delivered shortly after neuronal spiking are not strong enough to elicit spikes, which leads to longer time lags between post- and presynaptic neurons than for strong stimuli. This, on the other hand, reduces the contribution of long-term potentiation to the synaptic weight dynamics, which occurs for short positive time lags. We observe similar effects for NCR, SCR, and SNCR stimulation, compare Figures 3, 4, 7. Earlier studies also observed that weaker stimulation is more suitable for long-lasting effects. However, they considered spatial stimulation profiles and accounted for the fact that weaker stimulation affects a smaller tissue volume. For strong stimulation all neuronal subpopulations fired in response to the stimulation, and this in turn led to a weaker desynchronization and may not induce decoupling (Popovych and Tass, 2012; Lysyansky et al., 2013; Zeitler and Tass, 2015).

We point out that the stimulation duration is another relevant parameter; that is particularly important when it comes to the long-lasting effects of the stimulation. While stimulation parameters such as the number of stimulation sites, the stimulation amplitude, and the stimulation frequency determine whether the stimulation leads to a reduction of the mean synaptic weight, the time it takes to achieve a sufficient weight reduction and drive the network into the attractor of a stable desynchronized state depends on the actual rate of synaptic weight reduction. In Figure 8, stimulation is capable of inducing long-lasting desynchronization effects for all considered numbers of stimulation sites. However, the required stimulation duration to drive the network into the attractor of a desynchronized state varies by more than one order of magnitude. Thus, for too short stimulation duration, stimulation might be considered ineffective for inducing long-lasting effects even though it would be well capable of inducing such effects for a longer stimulation duration. This is particularly important as, e.g., the current parameter adjustment procedures for standard DBS focus on acute effects (Volkmann et al., 2006), which may compromise the potential long-lasting effects.

Our detailed analysis provides evidence that a reduction of spatial correlations between stimulus deliveries does not increase the robustness of long-lasting desynchronization effects with respect to changes of the stimulation frequency, see Figures 3, 4, 7. This is in line with the computational result from earlier studies where SCR stimulation was compared to CR stimulation with fixed sequence (Tass and Hauptmann, 2009). The authors reported that the weight reduction by SCR stimulation was weaker than CR stimulation. The authors further observed a strengthening of synaptic weights by strong SCR stimulation. However, stimulation parameters such as the stimulation frequency, the number of stimulation sites, as well as the stimulation duration were not varied systematically. In contrast, we provide a systematic comparison of the stimulation-induced synaptic weight dynamics, as well as the potential long-lasting desynchronization effects of CR and SCR stimulation using theoretical and computational analysis. We find that strong SCR stimulation leads to a different distribution of time lags between post- and presynaptic spikes than CR stimulation. Our theoretical analysis shows that this leads to a stronger contribution of long-term potentiation to the synaptic weight dynamics for high numbers of sites for the STDP function considered in the present paper, see Figure 2. The relative performance of CR and SCR stimulation may, however, differ for other STDP rules. In particular, Tass and Hauptmann (2009) considered a symmetric plasticity function. A detailed study of the performance of CR stimulation for different plasticity functions was provided in Kromer and Tass (2020).

Our systematic analysis provides evidence that adding random jitters to the stimulus delivery times might improve the parameter robustness of synaptic weight reduction and long-lasting desynchronization effects at intermediate stimulation frequencies. This was observed for strong stimulation, where spike times are determined by the stimulation pattern, see Figures 3, 4, and for weak stimulation see Figure 7. These results partly confirm the hypothesis that a reduction of spatio-temporal correlations in stimulus patterns increases parameter robustness of long-lasting effects, Kromer and Tass (2020); however, it also shows that this effect results mainly from a reduction of temporal correlations, while a reduction of spatial correlations by shuffling did not lead to an increase of parameter robustness. Motivated by the results of Kromer and Tass (2020), a NCR pattern of vibrotactile stimuli was studied computationally and in Parkinson's patients (Pfeifer et al., 2021). The observed results were similar as for a corresponding CR pattern, however, the number of patients included was too small to draw conclusions about the relative performance of both stimulation patterns. In Pfeifer et al. (2021) the stimulation mechanism was very different compared to the electrical stimulation used here and the jitter was only moderate. Besides this one study, the NCR pattern has not been studied before. However, other randomized versions of the classic CR pattern where considered in Tass and Hauptmann (2009) and Zeitler and Tass (2018). In Tass and Hauptmann (2009), a multi-site randomly timed reset (MRTR) pattern was considered in which activation times of individual sites were generated from a Poisson process, see Figure 8B in Tass and Hauptmann (2009). Desynchronization and synaptic weight reduction by MRTR were found to be robust with respect to variations of the stimulation amplitude, however, longer stimulation than for CR stimulation was required to achieve this effect. In Zeitler and Tass (2018) an uncorrelated multichannel noisy stimulation (UMNS) protocol was considered in which individual sites were activated at random times during each CR cycle, see Figure 1 in Zeitler and Tass (2018). The authors found that UMNS was able to reduce synaptic weights for the considered parameter combinations. In both studies, the authors did not vary the stimulation frequency and the number of stimulation sites systematically which makes a comparison to our results difficult. However, as MRTR and UMNS were obtained by temporal variation of stimulation times and shuffling, both patterns might be comparable to our SNCR stimulation, which was obtained by adding random jitters to CR with shuffled sequences. For strong and weak SNCR stimulation, we find that indeed weight reduction and long-lasting desynchronization effects are robust with respect to changes of the stimulation frequency and the number of stimulation sites, see Figures 3, 4, 7. Furthermore, the absolute mean rates of weight change in the region of slow stimulation and small numbers of stimulation sites are lower than those for CR stimulation. Consequently, although weight reduction and long-lasting effects are more robust with respect to parameter changes, longer stimulation is required to drive the network in the attractor of a stable desynchronized state.

For strong stimulation, our detailed analysis of the impact of random jitters on the performance of NCR and SNCR stimulation showed that jitters improve the parameter robustness of synaptic decoupling, as well as long-lasting desynchronization mainly in a limited range of stimulation frequencies, see Figure 6. Corresponding frequency ranges were denoted as $I_{1}$ (for NCR) and $I_{2}$ (for SNCR) and span the range of one to four times the frequency of the synchronous rhythm. In our network model, the latter is associated with a frequency of about 3.5 Hz. We find that $I_{1}$ is larger than $I_{2}$ indicating that shuffling reduces frequency robustness in the presence of random jitters. The range of these frequencies depends on the considered type of synaptic plasticity as well as the range of synaptic transmission delays. As argued above, the latter determines nonlinearities in the synaptic weight dynamics as a function of the stimulation frequency and the number of stimulation sites (Kromer et al., 2020) which, as we found in the present study, can be suppressed by inducing random jitters. We note that random jitters mainly lead to a continuous area in the parameter space where the stimulation leads to long-lasting desynchronization, see Figures 3, 4.

For weak stimulation, *A*_stim_ = 0.1, our results suggest that NCR with maximum variability of stimulus onset times (σ_CR_ = 1) may improve parameter robustness of long-lasting desynchronization effects in clinical studies for intermediate stimulation frequencies (one to four times the frequency of the pathological rhythm). In this range, random jitters expand the parameter region with effective long-lasting desynchronization toward high numbers of the stimulation site. However, the precise range depends on the underlying plasticity mechanism in the target area. As we have shown, the stimulation duration is a crucial factor, and a sufficiently long duration is needed to obtain long-lasting effects. Based on our results, one might be able to utilize the NCR stimulation in existing DBS electrodes to induce long-lasting effects.

In clinical trials, it is typically easier to vary stimulation parameters such as the stimulation pattern, the frequency, and the strength, rather than varying the number of stimulation sites. The latter are given by anatomical and physiological constraints of the target area. Hence, we studied long-lasting effects as a function of stimulation frequency and strength (see Figures 9, 10). In the limit of strong stimulation, *A*_stim_ → 1, the results approach the prediction of our theory (see Figure 2), i.e., we find a nonlinear dependence of long-lasting desynchronization effects on the stimulation frequency. However, for moderate stimulation strengths, 0.1 ≾ *A*_stim_ ≾ 0.5, we observe the highest degree of robustness with respect to variations of the stimulation frequency for all stimulation patterns (i.e., CR, SCR, NCR, and SNCR). Comparing CR and SCR with their noisy counterparts, NCR and SNCR, we find that including random jitters expands the parameter region with long-lasting desynchronization effects toward stronger stimulation.

Our results indicate that long-lasting desynchronization effects of stimulation with a small number of stimulation sites, e.g., *N*_s_ = 4, are robust with respect to changes of the stimulation frequency, even without random jitters. For larger numbers of stimulation sites (i.e, *N*_s_ = 8, 16, 24), the most robust long-lasting effects were observed for moderate stimulation strengths for all considered stimulation patterns. We found that jitter is particularly favorable for moderate to strong stimulation with larger numbers of stimulation sites.

Our promising results on the improved parameter robustness of long-lasting effects of NCR stimulation may trigger the question, whether randomized high-frequency stimulation (HF DBS) might be suitable to induce long-lasting therapeutic effects. To date, experimental studies on temporally randomized HF DBS mostly focus on acute intra-operative effects. Furthermore, it is still a matter of debate whether temporal randomization improves the acute effects of HF DBS. In a study by Brocker et al. (2013), it was reported that irregular HF DBS led to improved performance of PD patients in a finger-tapping task (Brocker et al., 2013). However, other studies reported that, in contrast to regular HF DBS, temporally randomized HF DBS was ineffective in providing symptom alleviation (Dorval et al., 2010; Birdno et al., 2012). To the best of our knowledge, no results on the long-lasting outcome of temporally randomized HF DBS have been presented to date.

In our recent study on CR stimulation, we observed that long-lasting effects are sensitive to the number of stimulation sites (Kromer et al., 2020). This suggest that the employment of recently developed DBS electrodes comprising large numbers of stimulation contacts (Krauss et al., 2020; Steigerwald et al., 2019) might actually require complicated parameter adjustment procedures. Here, we observe a similar parameter sensitivity for SCR stimulation, see Figures 3, 4, 7. However the long-lasting effects of stimulation patterns with random jitters, i.e., NCR and SNCR stimulation, are much more robust with respect to parameter changes, e.g., the number of stimulation sites used for stimulus delivery, see Figures 3, 4, 7. This provides evidence that NCR and SNCR stimulation might be more suitable for employing multisite stimulation electrodes for inducing long-lasting therapeutic effects. However, to date, there are no studies on the long-term use of such multisite stimulation electrodes available.

In DBS, the therapeutic outcome depends on the accurate placement of electrodes (Voges et al., 2002; Saint-Cyr et al., 2002). These electrodes typically have multiple stimulation contacts that, in some designs, can be activated independently (Krauss et al., 2020). Other designs allow for simultaneous co-activation of a subset of the stimulation sites to form an arbitrary number of co-activated subpopulations (Steigerwald et al., 2019). A common target area for DBS in Parkinson's disease is the STN (Krack et al., 2003). While the spatial extension of the entire STN is of the order of several millimeters to about one centimeter (Mavridis et al., 2013), the STN possesses a non-uniform somatotopic organization (Hartmann-von Monakow et al., 1978; Nambu et al., 1996; Nambu, 2011). In addition, based on incoming cortical projections, the STN is divided into sensorimotor, associative, and limbic parts (Nambu, 2011; Tewari et al., 2016). Depending on the patient's symptoms only a small portion of the STN is considered a suitable target area. For instance, a recent paper showed that while electrodes were placed such that a total of eight stimulation contacts where in the vicinity of the STN, only activation of a few contacts led to a reduction of pathological beta oscillations (Tamir et al., 2020). Thus, only a small number of contacts may be available for delivering multisite stimulation to the target area. In this context, it is encouraging that our results indicate that even multisite stimulation using just a small number of stimulation sites is capable of inducing long-lasting desynchronization effects that are robust with respect to variations of the stimulation frequency, *f*_CR_. However, our results also indicate that stimulation protocols with spatial randomization might be more suitable as they improve frequency-robustness for moderate to strong stimulations. Given the small anatomical target dimensions of, e.g., the STN, our results indicate that qualitatively different lead topologies comprising a larger number of smaller stimulation contacts with tighter spacing are not required to induce robust long-lasting desynchronization with the stimulation patterns studied here.

To conclude, multichannel CR stimulation has shown great promise for inducing long-lasting desynchronization and therapeutic effects when delivered through DBS electrodes (Tass et al., 2012b; Adamchic et al., 2014; Wang et al., 2016) or through vibrotactile fingertip stimulators (Pfeifer et al., 2021). Our results suggest that the robustness of long-lasting effects with respect to changes of the stimulation frequency and other parameters might be increased by adding jitters to the stimulus delivery times. In contrast, shuffling the sequence of stimulus deliveries does not increase parameter robustness. In our model, acute after-effects of stimulation are strongly correlated with long-lasting effects. This suggests that acute after-effects might be suitable for predicting long-lasting effects of the stimulation. We hope that our results will lead to more clinical studies on stimulation protocols with random jitters to improve parameter adjustment procedures for brain stimulation as a treatment for neurological disorders.

## Data Availability Statement

The original contributions generated for the study are included in the article/supplementary material, further inquiries can be directed to the corresponding author.

## Author Contributions

AKN, JK, and PT conceived the idea, designed the study, interpreted the results, and wrote the manuscript. AKN performed the numerical simulations and analysis. JK developed the theory. All authors contributed to the article and approved the submitted version.

## Funding

We gratefully acknowledge support of this study by Boston Scientific Neuromodulation (Stanford Project 127674) and the Foundation for OCD Research (New Venture Fund, 011665-2020-08-01).

## Conflict of Interest

PT works as a consultant for Boston Scientific Neuromodulation. The remaining authors declare that the research was conducted in the absence of any commercial or financial relationships that could be construed as a potential conflict of interest.

## Publisher's Note

All claims expressed in this article are solely those of the authors and do not necessarily represent those of their affiliated organizations, or those of the publisher, the editors and the reviewers. Any product that may be evaluated in this article, or claim that may be made by its manufacturer, is not guaranteed or endorsed by the publisher.

## Appendix

### Neuronal Network Model

In our LIF model the spiking occurs when the membrane potential *v*_*i*_ crosses a dynamic threshold potential $v_{i}^{\text{th}}$; where the membrane and the threshold potentials of neuron *i* obeys:

(A1)
$$\begin{array}{l} C_{i}\frac{\text{d}v_{i}}{\text{d}t}=g_{\text{leak}}\left(v_{\text{rest}}-v_{i}\right)+I_{i}^{\text{syn}}\left(t\right)+I_{i}^{\text{stim}}\left(t\right)+I_{i}^{\text{noise}}\left(t\right).\\ \tau_{\text{th}}\frac{\text{d}v_{i}^{\text{th}}}{\text{d}t}=\left(v_{\text{rest}}^{\text{th}}-v_{i}^{\text{th}}\right). \end{array}$$

Here $I_{i}^{\text{syn}}\left(t\right)$ is the synaptic input current, $I_{i}^{\text{stim}}\left(t\right)$ the stimulation current, $I_{i}^{\text{noise}}\left(t\right)$ the noisy input current, *C*_*i*_ is the membrane capacitance, τ_th_ is the threshold time constant and $v_{\text{rest}}^{\text{th}}$ the stationary threshold potential, *v*_rest_ the resting potential, and *g*_leak_ is the leak conductance.

Spike times are recorded at each threshold crossing. After a spike, the membrane potential is set to *v*_spike_ for a time period of *t*_spike_; then, we perform an instantaneous reset: *v*_*i*_→*v*_reset_ and $v_{i}^{\text{th}}\to v_{\text{spike}}^{\text{th}}$.

We use the same parameters as in Kromer et al. (2020) and Khaledi-Nasab et al. (2021b): *v*_rest_ = −38 mV, τ_th_ = 5 ms, $v_{\text{rest}}^{\text{th}}=-40$ mV, *t*_spike_ = 1 ms, *v*_spike_ = 20 mV, *V*_reset_ = −67 mV, and *g*_leak_ = 0.02 mS/cm^2^. The capacitances *C*_*i*_ follow a normal distribution with mean value of 〈*C*_*i*_〉 = 3 μF/cm^2^ and standard deviation of 0.05〈*C*_*i*_〉.

Each neuron *i* receives noisy input, $I_{i}^{\text{noise}}$, which is obtained by feeding presynaptic Poisson spike trains with firing rate *f*_noise_ = 20 Hz into excitatory synapses (Ebert et al., 2014)

(A2)
$$\begin{array}{l} I_{i}^{\text{noise}}=g_{i}^{\text{noise}}\left(v_{\text{syn}}-v_{i}\right),\\ \tau_{\text{syn}}\frac{\text{d}g_{i}^{\text{noise}}}{\text{d}t}=-g_{i}^{\text{noise}}+D\underset{k_{i}}{\sum }\delta \left(t_{k_{i}}^{i}-t\right). \end{array}$$

Here the noise intensity is controlled by the parameter *D* = 0.026 *mS*/*cm*^2^ scaling the strength of the Poisson input. *v*_syn_ = 0 mV is the synaptic reversal potential, τ_syn_ = 1 ms the synaptic time scale, and $g_{i}^{\text{noise}}\left(t\right)$ the synaptic conductance.

$I_{i}^{\text{syn}}\left(t\right)$ is the excitatory synaptic input to neuron *i*, and it is given by

(A3)
$$\begin{array}{l} I_{i}^{\text{syn}}=g_{i}^{\text{syn}}\left(v_{\text{syn}}-v_{i}\right),\\ \tau_{\text{syn}}\frac{\text{d}g_{i}^{\text{syn}}}{\text{d}t}=-g_{i}^{\text{syn}}+\frac{\kappa }{N}\underset{j\in G_{i}}{\sum }w_{ji}\underset{l^{j}}{\sum }\delta \left(t-t_{l^{j}}^{j}-t_{\text{d}}\right), \end{array}$$

where κ = 8 mS/cm^2^ is the coupling strength, $g_{i}^{\text{syn}}$ is the synaptic conductance, *w*_*ji*_ ∈ [0, 1] is the weight of the synapses between presynaptic neuron *j* and postsynaptic neuron *i*. The first sum runs over all presynaptic neurons, and the second sum runs over the presynaptic spikes of neuron *j*. We consider homogeneous synaptic delays of *t*_d_ = 3 ms.
